# Jiawei Kongsheng Zhenzhong Pill: marker compounds, absorption into the serum (rat), and Q-markers identified by UPLC-Q-TOF-MS/MS

**DOI:** 10.3389/fphar.2024.1328632

**Published:** 2024-02-05

**Authors:** Qiaolan Wu, Chunxue Ou, Jiayun Wang, Xiaolin Wu, Zu Gao, Yue Zhao, Guangying Lu, Zhichun Wu, Huayun Yu

**Affiliations:** ^1^ College of Traditional Chinese Medicine, Shandong University of Traditional Chinese Medicine, Jinan, China; ^2^ Experimental Center, Shandong University of Traditional Chinese Medicine, Jinan, China; ^3^ Shandong Provincial Co-innovation Center of Classic TCM Formula, Jinan, China

**Keywords:** Jiawei Kongsheng Zhenzhong Pill, compounds, Chinese herbal prescription, ultra-performance liquid chromatography quadrupole time-of-flight mass tandem spectrometry, quality marker, neuroprotective effect, pharmacodynamic material basis

## Abstract

**Background:** The Jiawei Kongsheng Zhenzhong pill (JKZP), a Chinese herbal prescription comprised of eight Chinese crude drugs, has been historically employed to treat neurological and psychological disorders. Nevertheless, the ambiguous material basis severely hindered its progress and application.

**Purpose:** The current study aimed to establish a rapid analytical method for identifying the chemical components of the JKZP aqueous extract and the components absorbed into the rat serum to investigate the quality markers (Q-markers) responsible for the neuroprotective effects of JKZP.

**Methods:** The qualitative detection of the chemical components, prototype components, and metabolites of the aqueous extracts of JKZP, as well as the serum samples of rats that were administered the drug, was performed using the ultra-performance liquid chromatography- quadrupole time-of-flight tandem mass spectrometry (UPLC-Q-TOF-MS/MS) technology. This analysis combined information from literature reports and database comparisons. Moreover, the study was conducted to anticipate the potential Q-markers for the neuroprotective effects of JKZP based on the “five principles” of Q-marker determination.

**Results:** A total of 67 compounds and 111 serum components (comprising 33 prototypes and 78 metabolites) were detected and identified. Combining the principles of quality transmission and traceability, compound compatibility environment, component specificity, effectiveness, and measurability, the study predicted that five key compounds, namely, senkyunolide H, danshensu, echinacoside, loganin, and 3,6′-disinapoyl sucrose, may serve as potential pharmacological bases for the neuroprotective effects of JKZP.

**Conclusion:** To summarize, the UPLC-Q-TOF-MS/MS technique can be employed to rapidly and accurately identify compounds in JKZP. Five active compounds have been predicted to be the Q-markers for the neuroprotective effects of JKZP. This discovery serves as a reference for improving quality, advancing further research and development, and utilizing Chinese herbal prescriptions.

## 1 Introduction

The Jiawei Kongsheng Zhenzhong pill (JKZP) was derived from the Kongsheng Zhenzhong pill as documented in the ancient medical classic “Thousand Golden Prescriptions.” The original prescription, consisting of *Chinemys reevesii* (Gray) (tortoise plastron; also named Guijia in Chinese), *Os Draconis* (*Fossilia Ossia Mastodi*) (dragon bones; also named Longgu in Chinese), *Polygalae tenuifolia* Willd (Radix Polygalae; also named Yuanzhi in Chinese), and *Acorus tatarinowii* Schott (Acorus Tatarinowii; also named Shichangpu in Chinese), manifested effects in tonifying the kidneys, tranquilizing the heart, enhancing wisdom, and soothing the mind. It was also esteemed as a conventional prescription for heightening wisdom and enlightenment. Consequently, its primary objective was to address conditions such as insomnia, amnesia, Alzheimer's disease, vascular cognitive impairment, depression, pediatric hyperactivity disorder, and similar ailments clinically (Rui and Yue, 2012; Hou et al., 2021; Chen et al., 2022). In accordance with traditional Chinese medicine (TCM) theory and clinical experience, *Salvia miltiorrhiza* Bunge (Salviae Miltiorrhizae; also named Danshen in Chinese), *Ligusticum sinense* ‘Chuanxiong’ (Chuanxiong Rhizoma; also named Chuanxiong in Chinese), *Cornus officinalis* Sieb. et Zucc. (Corni Fructus; also named Shanzhuyu in Chinese), and *Cistanche deserticola* Ma (Cistanches Herba; also named Roucongrou in Chinese) were incorporated into the original prescription to reinforce the effects of kidney and marrow tonification, blood circulation activation, blood stasis removal, and depression relief. Its principal application targeted symptoms associated with renal essence deficiency and blood stasis. What is more, it has demonstrated notable therapeutic effectiveness in addressing vascular cognitive impairment and post-stroke depression, exhibiting a commendable neuroprotective effect (Yu et al., 2013; Wu et al., 2015; Pang et al., 2016; Song, 2022; Yu et al., 2016; Zhou, 2022). However, the components of JKZP are relatively multifarious, and the precise pharmacological basis for its neuroprotective effect requires further elucidation. The compositions and proportions of JKZP are shown in Table 1.

**TABLE 1:** Composition of Jiawei Kongsheng Zhenzhong pill (JKZP).

English name	Botanical name	Chinese name	Part used	Proportion
Tortoise plastron	*Chinemys reevesii* (Gray)	Guijia	Shell	6
Dragon bones	*Os Draconis* (*Fossilia Ossia Mastodi*)	Longgu	Bone fossils of big mammals	6
Radix Polygalae	*Polygalae tenuifolia* Willd	Yuanzhi	Root	3
Acorus Tatarinowii	*Acorus tatarinowii* Schott	Shichangpu	Root	3
Salviae Miltiorrhizae	*Salvia miltiorrhiza* Bunge	Danshen	Root and rhizome	5
Chuanxiong Rhizoma	*Ligusticum sinense* ‘Chuanxiong'	Chuanxiong	Rhizome	4
Corni Fructus	*Cornus officinalis* Sieb. et Zucc	Shanzhuyu	Sarcocarp	5
Cistanches Herba	*Cistanche deserticola* Ma	Roucongrou	Succulent stem	4

The plant name has been checked and confirmed with the WFO Plant List and Plants of the World Online | Kew Science.

Over the past few decades, an increasing number of studies have reported the components in the aqueous extract and the serum of the single plant drug in JKZP (Li et al., 2008; Yun et al., 2018; Hou, 2019; Qu et al., 2020; Yang et al., 2020; Zhao et al., 2020), which are aligned with prior research, and certain components that exhibit noteworthy neuroprotective activities (Feng et al., 2017; Wang et al., 2020a; Tang X. et al., 2022; Lu et al., 2022). Nevertheless, research reports regarding the analysis and identification of compounds in the whole prescription of JKZP require further elaboration. Quality markers (Q-markers) are compounds intricately linked to the pharmacological properties of herbs, particularly their effectiveness and measurability. They served for quality control in single herbs and Chinese herbal prescriptions, revealing potential pharmacological substance foundations, thus facilitating further research, development, and utilization (Jiang et al., 2023). The widely utilized technology for the qualitative detection of compounds in TCM is ultra-performance liquid chromatography quadrupole time-of-flight tandem mass spectrometry (UPLC-Q-TOF-MS/MS). This technology is distinguished by its robust separation capability, high sensitivity, and convenience. The components in the sample are ionized, resulting in ions with a certain charge and different mass numbers. Different ions have different motion behaviors in the electromagnetic field. The mass analyzer is used to separate ions according to the different mass-to-charge ratios (*m/z*), obtaining the mass spectra in the order of the mass-to-charge ratios and then comparing these with the database, which can be used for the identification of the properties of the compounds (Chen et al., 2023a).

Hence, this study conducted preliminary qualitative analyses and identifications of the aqueous extract of JKZP and JKZP-containing serum of rats using the UPLC-Q-TOF-MS/MS technique. Concurrently, leveraging the neuroprotective effect of JKZP, potential Q-markers were forecasted following the ‘five principles’ of Q-marker determination. This study sought to contribute insights and foundations for conducting fundamental research on the pharmacological substances of Chinese herbal prescriptions and improving the criteria for quality control.

## 2 Materials and methods

### 2.1 Apparatus

Waters H-Class UPLC (Waters, United States), AB Sciex Triple TOF^®^ 4600 high-resolution mass spectrum (SCIEX, United States), KQ-300 BD ultrasonic cleaning instrument (Kunshan Ultrasonic Instrument, China), Sigma 3K15 high-speed centrifuge (Sigma, United States), LNG-T98 centrifugal concentration dryer (Taicang Huamei, China), and R583S small animal anesthesia machine (RWD, China) were used for this study.

### 2.2 Reagents and materials

The reagents and materials used for the study involved Chinese medicinal decoction pieces of JKZP (tortoise plastron, lot: 19081001; dragon bones, lot: 20200201; Radix Polygalae, lot: 20113001; Acorus Tatarinowii, lot: 20103001; Salviae Miltiorrhizae, lot: 20121401; Chuanxiong Rhizoma, lot: 20092103; Corni Fructus, lot: 20040801; Cistanches Herba, lot: 21021904), which were purchased from Shandong Bokang TCM Decoction Pieces Co. Ltd and identified as genuine, acetonitrile (MS pure, I1133829105, Merck company), methanol (MS pure, I1139035113, Merck company), formic acid (MS pure, Y6170039, CNW company), purified water (20221110C, Guangzhou Watsons Food and Beverage Co., Ltd.), and isoflurane (R510-22-10, Shenzhen Ruiwode Life Technology Co., Ltd.).

### 2.3 Preparation of the JKZP aqueous extract solution

To ensure the consistency of JKZP, the quality of each herb was evaluated before use, and the extraction of the decoction followed the standardized procedures specified in the “Pharmacopoeia of the People's Republic of China.” Chuanxiong Rhizoma (120 g) and Acorus Tatarinowii (90 g) were broken and soaked in a distillation flask for 30 min, and then volatile oil was extracted by distillation for 6 h. The volatile oil was taken out, and the filtrate and dregs were stored temporarily. After soaking for 60 min, the tortoise plastron (180 g) and dragon bones (180 g) were broken into pieces and decocted for 30 min and then added to the other medicines (Radix Polygalae, 90 g; Salviae Miltiorrhizae, 150 g; Corni Fructus, 150 g; and Cistanches Herba, 120 g) and dregs of Chuanxiong Rhizoma and Acorus Tatarinowii, and the entire mixture was then sequentially decocted for 45 min. The second decoction was made, and the filtrate was combined and filtered. The filtrate was concentrated by spinning in a water bath at 65°C, the volatile oil was combined, and the concentration of the drug aqueous extract solution was adjusted to 3.6 g mL^−1^ and stored at 4°C.

### 2.4 Animals, drug administration, and serum samples' pretreatment

Healthy male SD rats of SPF grade with a body weight of 250 ± 10 g were purchased from Beijing Vital River Laboratory Animal Technology Co., Ltd., under animal production license no. SCXK (Beijing) 2021-0011. They were raised in an environment with a temperature of 20–26°C, relative humidity of 55 ± 10%, and light/darkness cycle of 12 h each, with *ad libitum* access to food and water. The animal handling during this experiment adhered to China's “Regulations on the Management of Laboratory Animals” and the relevant regulations of the Ethics Committee of the Laboratory Animal Center of Shandong University of Traditional Chinese Medicine. The study received ethical approval, with the number SDUTCM20221021004.

A total of 20 rats were randomly allocated into a JKZP-containing serum group and a blank serum group, with 10 rats in each group. In the JKZP-containing serum group, rats received 56.7 g kg^−1^ d^−1^ of JKZP through gavage (calculated based on an adult body weight of 60 kg, and five times the equivalent clinical dose) for five consecutive days. The blank serum group received an equal volume of saline.

Within 1 h post final intragastric administration, continuous anesthesia was induced by inhaling 3% isoflurane. Blood was drawn from the abdominal aorta and left at room temperature for 2 h. Subsequently, serum separation was performed by centrifugation at 3000 rpm min^−1^ for 15 min. The complement was inactivated using a constant temperature water bath set at 56°C for 30 min. The serum from the same group was pooled to minimize individual variations, sub-packaged, and stored at −80°C for subsequent use.

### 2.5 Preparation of the sample solution

For the JKZP aqueous extract sample, 1 mL of the JKZP aqueous extract was placed in a centrifuge tube, and 2 mL of 20% methanol was added, then the supernatant was collected after centrifugation at 12,000 rpm min^−1^ for 15 min. For the serum sample, 2 mL of the rat blank serum and 2 mL of JKZP-containing serum were taken separately. Thrice the volume of methanol (mass spectrometry grade) was added to the precipitate proteins. The mixture was thoroughly mixed and stored at 4°C for 20 min, followed by centrifugation to obtain the supernatant. The supernatant was concentrated, dried by centrifugation, and stored at −80°C. The residue was dissolved in 200 μL of 50% methanol before analysis, thoroughly mixed, and then centrifuged to obtain the supernatant.

### 2.6 Chromatographic and mass spectrometry conditions

The chromatography analysis was performed using Waters^®^ CORTECS^®^ UPLC^®^ T3 (1.6 µm, 2.1 mm × 100 mm) at 30°C. The mobile phase consisted of acetonitrile (A) and 0.1% formic acid in water (B). The gradient elution with the following program was carried out as follows: 0–3 min, 0% A; 3–7 min, 0%–5% A; 7–30 min, 5%–13% A; 30–55 min, 13%–25% A; 55–67 min, 25%–40% A; 67–72 min, 40%–95% A; 72–75 min, 95% A; 75–75.1 min, 95%–0% A; 75.1–78 min, 0% A. The flow rate was set at 0.3 mL min^−1^, the detection wavelength was 190–400 nm, and the injection volume was 2–5 μL. The MS detection was applied in an ESI-negative/positive ion mode.

### 2.7 Data analysis

Data analysis was conducted using the PeakView 1.2 software. MS data were preferentially matched with the Natural Products HR-MS/MS Spectral Library 1.0 database for identification by comparison with the controls. Compounds were initially screened based on the data of each peak and subsequently confirmed using the primary and secondary information of each peak. Subsequently, considering the preliminary analysis of the identified components using the UPLC-Q-TOF-MS/MS technique, potential Q-markers for the neuroprotective effect of JKZP were predicted following the five principles of Q-marker determination.

## 3 Results

### 3.1 Acquisition and identification of the UPLC-Q-TOF-MS/MS chromatogram of the JKZP aqueous extract

**FIGURE 1:**
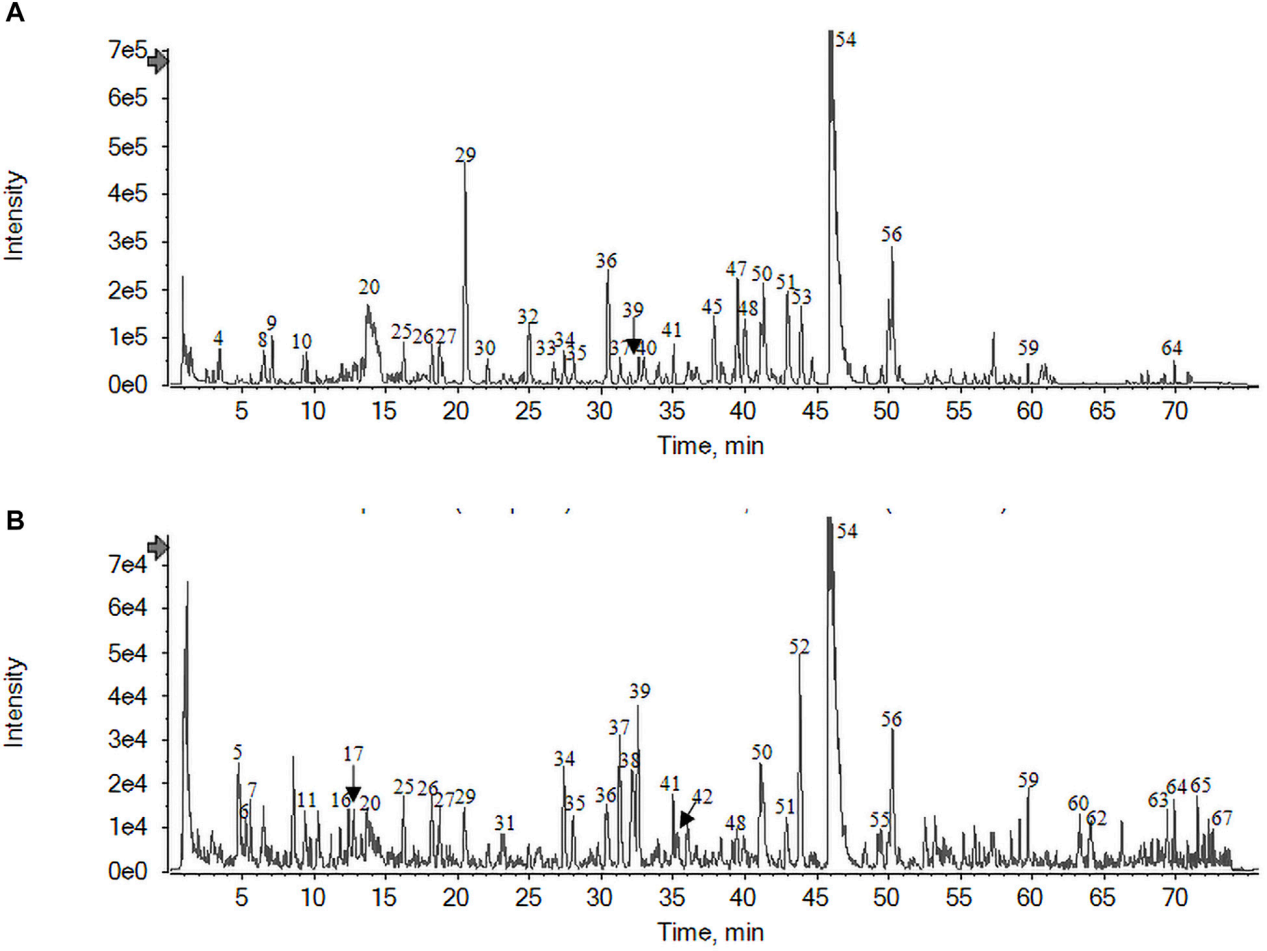
Total ion flow diagram of the JKZP aqueous extract by UPLC-HRMS: **(A)** negative ion modes; **(B)** positive ion modes.

Under the aforementioned chromatographic and MS conditions, total ion flow diagrams of the JKZP aqueous extract were, respectively, collected in positive and negative ion modes. As illustrated in Figure 1A, B, the initial observation of the images revealed that the total ion flow map was clear, enabling data analysis. The results exhibited a high degree of reliability. More narrowly, the peak of gallic acid merged in 3.44 min, labeled as No. 4 in Figure 1A, while the peak of danshensu appeared in 7.05 min, described as No. 9 also in Figure 1A.

Utilizing the multistage MS information of the samples, coupled with the natural product high-resolution MS database and relevant literature, the ion peaks in the total ion flow diagram of the JKZP samples were identified. As depicted in Table 2, a total of 12 classes and 67 compounds were identified in the whole ion flow diagram of JKZP samples. These included 18 phenolic acids, 13 iridoid glycosides, 10 oligosaccharide esters, four each of amino acids, phenylethanoid glycosides, phthalides, and diterpene quinones, three each of xanthones and nucleosides, two phenylpropanoids, and one each of organic acid and cyclic peptide.

**TABLE 2:** Main component identification results of the JKZP aqueous extract.

No.	Time (min)	Adduction	*m/z* Actual value	Molecular formula	Molecular weight	Component	MS/MS data	Classification	Source
1	2.10	[M + H]^+^	132.1014	C_6_H_13_NO_2_	131.09	L-Leucine	86.0946; 69.0677; 56.0481	Amino acids	Guijia
2	2.35	[M + H]^+^	182.0808	C_9_H_11_NO_3_	181.07	L-Tyrosine	136.0765; 119.0494; 107.0490; 91.0542	Amino acids	Guijia
3	2.50	[M − H]^−^	243.0636	C_9_H_12_N_2_O_6_	244.07	Uridine	243.0633; 200.0583; 152.0368; 110.0257; 82.0299	Nucleosides	—
4	3.44	[M − H]^−^	169.0147	C_7_H_6_O_5_	170.02	Gallic acid	169.0145; 125.0248; 97.0293; 79.0189	Phenolic acids	Shanzhuyu
5	4.73	[M + H]^+^	268.1041	C_10_H_13_N_5_O_4_	267.10	Adenosine	268.1030; 136.0611; 119.0346	Nucleosides	—
6	5.23	[M + H]^+^	166.0851	C_9_H_11_NO_2_	165.08	Phenylalanine	120.0803; 103.0538; 91.0532; 77.0378	Amino acids	Guijia
7	5.56	[M − H]^−^	282.0840	C_10_H_13_N_5_O_5_	283.09	Guanosine	282.0872; 150.0428; 133.0158	Nucleosides	—
8	6.47	[M − H]^−^	361.0780	C_14_H_18_O_11_	362.08	7-O-Galloyl-D-sedoheptulose	271.0468; 211.0257; 169.0149; 124.0171	Phenolic acids	Shanzhuyu
9	7.05	[M − H]^−^	197.0458	C_9_H_10_O_5_	198.05	Danshensu	179.0351; 135.0451; 123.0452; 72.9931	Phenolic acids	Danshen
10	9.22	[M − H]^−^	137.0252	C_7_H_6_O_3_	138.03	Protocatechuic aldehyde	137.0250; 136.0171; 108.0221; 92.0269	Phenolic acids	Danshen
11	9.36	[M + H]^+^	205.0968	C_11_H_12_N_2_O_2_	204.09	L-Tryptophan	188.0696; 170.0583; 146.0592; 118.0644	Amino acids	—
12	10.16	[M − H]^−^	353.0886	C_16_H_18_O_9_	354.10	Neochlorogenic acid	353.0881; 191.0565; 179.0356; 135.0455	Phenolic acids	Chuanxiong
13	11.29	[M + FA − H]^−^	391.1241	C_15_H_22_O_9_	346.13	Aucubin	391.1278; 345.1187; 183.0653; 165.0551; 161.0456; 153.0556	Iridoid glycosides	Shanzhuyu, Roucongrong
14	11.93	[M − H]^−^	375.1290	C_16_H_24_O_10_	376.14	Loganic acid	375.1306; 213.0774; 169.0879; 151.0768	Iridoid glycosides	Shanzhuyu
15	12.23	[M − H]^−^	461.1296	C_19_H_26_O_13_	462.14	Sibiricose A3	461.1310; 299.0780; 281.0670; 239.0558; 179.0352; 137.0242	Oligosaccharide esters	Yuanzhi
16	12.39	[M-H]^-^	567.1950	C_23_H_36_O_16_	568.20	Cornusdiglycoside G	567.1402; 405.1398; 285.0977; 243.0865	Iridoid glycosides	Shanzhuyu
17	12.76	[M + FA − H]^−^	613.2013	C_23_H_36_O_16_	568.20	Cornusdiglycoside H	405.1417; 243.0883; 179.0568	Iridoid glycosides	Shanzhuyu
18	12.98	[M + FA − H]^−^	451.1446	C_17_H_26_O_11_	406.15	7α-Morroniside	405.1428; 243.0883; 179.0568; 155.0345; 141.0559	Iridoid glycosides	Shanzhuyu
19	13.39	[M − H]^−^	311.0408	C_13_H_12_O_9_	312.05	Caftaric acid	311.0412; 179.0359; 149.0098; 135.0457	Phenolic acids	Danshen
20	13.96	[M + FA − H]^−^	451.1466	C_17_H_26_O_11_	406.15	Morroniside	451.1487; 405.1418; 243.0883; 179.0568; 155.0360; 141.0564	Iridoid glycosides	Shanzhuyu
21	14.32	[M − H]^−^	353.0868	C_16_H_18_O_9_	354.10	Chlorogenic acid	353.0845; 191.0555; 171.0298; 161.0244	Phenolic acids	Chuanxiong
22	15.45	[M − H]^−^	389.1081	C_16_H_22_O_11_	390.12	Secoxyloganic acid	389.1080; 345.1184; 209.0436; 165.0548	Iridoid glycosides	Shanzhuyu
23	15.72	[M − H]^−^	353.0881	C_16_H_18_O_9_	354.10	Cryptochlorogenic acid	353.0920; 191.0568; 179.0352; 173.0462; 135.0456	Phenolic acids	Chuanxiong
24	15.98	[M + FA − H]^−^	475.1490	C_19_H_26_O_11_	430.15	Polygalatenoside A	475.0942; 429.1415; 307.1024; 255.1052; 121.0298	Oligosaccharide esters	Yuanzhi
25	16.23	[M − H]^−^	517.1554	C_22_H_30_O_14_	518.16	Sibiricose A5	517.1572; 337.0921; 193.0504; 175.0398; 160.0162	Oligosaccharide esters	Yuanzhi
26	18.17	[M − H]^−^	547.1685	C_23_H_32_O_15_	548.17	Sibiricose A1	547.1651; 341.1066; 223.0605; 205.0499; 190.0261	Oligosaccharide esters	Yuanzhi
27	18.74	[M + FA − H]^−^	403.1244	C_16_H_22_O_9_	358.13	Sweroside	403.1255; 357.1185; 195.0667; 125.0249	Iridoid glycosides	Shanzhuyu
28	18.94	[M + FA − H]^−^	433.1372	C_17_H_24_O_10_	388.14	Cornin	433.1362; 387.1318; 255.0771; 123.0451	Iridoid glycosides	Shanzhuyu
29	20.51	[M + FA − H]^−^	435.1526	C_17_H_26_O_10_	390.15	Loganin	435.1509; 227.091; 127.0398; 101.0243	Iridoid glycosides	Shanzhuyu
30	22.11	[M − H]^−^	515.1219	C_25_H_24_O_12_	516.13	Isochlorogenic acid A	515.1213; 353.0885; 335.0774; 191.0568; 179.0355; 135.0454	Phenolic acids	Chuanxiong
31	23.17	[M − H]^−^	193.0514	C_10_H_10_O_4_	194.06	Ferulic acid	178.0274; 134.0377; 133.0296	Phenolic acids	Chuanxiong, Shichangpu
32	24.95	[M − H]^−^	505.1595	C_21_H_30_O_14_	506.16	Logmalicid A	505.1585; 487.1456; 389.1452; 227.0925; 127.0401	Iridoid glycosides	Shanzhuyu
33	26.68	[M − H]^−^	505.1571	C_21_H_30_O_14_	506.16	Logmalicid B	505.1575; 487.1459; 227.0926; 127.0393	Iridoid glycosides	Shanzhuyu
34	27.40	[M − H]^−^	537.1282	C_24_H_26_O_14_	538.13	Sibiricaxanthone A	537.1237; 417.0809; 405.0818; 387.0702; 315.0493; 285.0392; 267.0285	Xanthones	Yuanzhi
35	28.07	[M + FA − H]^−^	607.1875	C_24_H_34_O_15_	562.19	Glomeratose A	607.1894; 561.1838; 323.0973; 237.0769; 193.0870	Oligosaccharide esters	Yuanzhi
36	30.25	[M − H]^−^	785.2530	C_35_H_46_O_20_	786.26	Echinacoside	785.2515; 623.2213; 461.1679; 161.0251	Phenylethanoid glycosides	Roucongrong
37	31.28	[M − H]^−^	567.1409	C_25_H_28_O_15_	568.14	Polygalaxanthone III	567.1409; 359.1523; 345.0630; 329.1409	Xanthones	Yuanzhi
38	32.17	[M − H_2_O + H]^+^	207.1021	C_12_H_16_O_4_	224.10	Senkyunolide I	207.1050; 189.0922; 161.0962; 133.0652; 91.0534	Phthalides	Chuanxiong
39	32.59	[M − H]^−^	567.1383	C_25_H_28_O_15_	568.14	Polygalaxanthone XI	567.1402; 345.0627; 315.0513; 272.0317	Xanthones	Yuanzhi
40	32.96	[M − H]^−^	187.0981	C_9_H_16_O_4_	188.10	Azelaic acid	187.0990; 125.0980; 97.0665	Organic acids	Yuanzhi
41	35.01	[M − H]^−^	799.2696	C_36_H_48_O_20_	800.27	Cistanoside A	799.2669; 623.2213; 605.2162; 477.1456; 175.0396	Phenylethanoid glycosides	Roucongrong
42	35.30	[M − H_2_O + H]^+^	207.1020	C_12_H_16_O_4_	224.10	Senkyunolide H	207.1023; 189.0909; 161.0968; 133.0636; 91.0531	Phthalides	Chuanxiong
43	36.09	[M − H]^−^	537.1048	C_27_H_22_O_12_	538.11	Salvianolic acid H	339.0518; 295.0618; 185.0246	Phenolic acids	Danshen
44	36.36	[M − H]^−^	623.1979	C_29_H_36_O_15_	624.21	Acteoside	623.2013; 461.1676; 161.0245	phenylethanoid glycosides	Roucongrong
45	37.83	[M − H]^−^	417.0819	C_20_H_18_O_10_	418.09	Salvianolic acid D	417.0800; 197.0449; 175.0396; 157.0284	Phenolic acids	Danshen
46	38.50	[M − H]^−^	623.2010	C_29_H_36_O_15_	624.21	Isoacteoside	623.2016; 461.1695; 161.0253	Phenylethanoid glycosides	Roucongrong
47	39.46	[M − H]^−^	541.1575	C_24_H_30_O_14_	542.16	Cornuside	541.1570; 169.0145; 125.0246	Iridoid glycosides	Shanzhuyu
48	39.97	[M − H]^−^	359.0787	C_18_H_16_O_8_	360.08	Rosmarinic acid	197.0457; 179.0351; 161.0246; 133.0297	Phenolic acids	Danshen
49	41.15	[M − H]^−^	753.2277	C_34_H_42_O_19_	754.23	3,6′-Disinapoyl sucrose	753.2296; 547.1709; 519.0969; 205.0521; 190.0299	Oligosaccharide esters	Yuanzhi
50	41.31	[M − H]^−^	717.1509	C_36_H_30_O_16_	718.15	Salvianolic acid E	717.1437; 519.0917; 339.0501; 321.0390	Phenolic acids	Danshen
51	42.95	[M − H]^−^	537.1078	C_27_H_22_O_12_	538.11	Lithospermic acid	493.1141; 295.0617; 203.0357; 185.0251	Phenolic acids	Danshen
52	43.81	[M + H]^+^	679.5136	C_36_H_66_N_6_O_6_	678.50	Cyclohexaleucyl	679.5142; 661.5020; 336.2274; 209.1656	Cyclic peptides	—
53	43.93	[M − H]^−^	681.2049	C_31_H_38_O_17_	682.21	Tenuifoliside A	681.2066; 443.1200; 281.0675; 179.0359; 137.0249	Oligosaccharide esters	Yuanzhi
54	45.99	[M − H]^−^	717.1504	C_36_H_30_O_16_	718.15	Salvianolic acid B	717.1506; 519.0958; 339.0527; 321.0414	Phenolic acids	Danshen
55	49.98	[M − H]^−^	493.1167	C_26_H_22_O_10_	494.12	Salvianolic acid A	493.1180; 295.0626; 185.0253	Phenolic acids	Danshen
56	50.25	[M − H]^−^	717.1480	C_36_H_30_O_16_	718.15	Salvianolic acid Y	717.1532; 519.0972; 339.0518; 321.0407	Phenolic acids	Danshen
57	52.55	[M − 2H]^2−^	747.2313	C_67_H_84_O_38_	1496.46	Tenuifoliose L	1203.3663; 747.2210; 674.1978; 145.0300	Oligosaccharide esters	Yuanzhi
58	52.65	[M + FA − H]^−^	711.2196	C_31_H_38_O_16_	666.22	3-O-[(E)-3,4,5-Trimethoxycinnamoyl]-β-D-fructofuranosyl-(2→1)-(6-O-benzoyl)-α-D-glucopyranoside	665.2203; 543.1774; 427.1259; 237.0777	Oligosaccharide esters	Yuanzhi
59	59.69	[M − H]^−^	1379.4160	C_62_H_76_O_35_	1380.42	Tenuifoliose A	—	Oligosaccharide esters	Yuanzhi
60	63.29	[M + H]^+^	209.1178	C_12_H_16_O_3_	208.11	Cis-β-asarone	209.1154; 194.0934; 179.0698; 151.0748; 103.0533	Phenylpropanoids	Shichangpu
61	63.69	[M + H]^+^	179.1069	C_11_H_14_O_2_	178.10	Methyl eugenol	164.0834; 151.0757; 149.0586; 121.0636; 91.0532	Phenylpropanoids	Shichangpu
62	64.03	[M + H]^+^	193.1232	C_12_H_16_O_2_	192.12	Senkyunolide A	175.1095; 147.1153; 137.0583; 91.0538	Phthalides	Chuanxiong
63	69.38	[M + H]^+^	191.1070	C_12_H_14_O_2_	190.10	Ligustilide	191.1060; 173.0955; 115.0532; 91.0532	Phthalides	Chuanxiong
64	69.87	[M + H]^+^	315.1610	C_19_H_22_O_4_	314.15	Neocryptotanshinone	297.1497; 253.1605; 137.0918; 223.1129; 211.1124	Diterpene quinones	Danshen
65	71.50	[M + H]^+^	297.1496	C_19_H_20_O_3_	296.14	Cryptotanshinone	297.1494; 279.1389; 267.1006; 251.1433	Diterpene quinones	Danshen
66	71.58	[M + H]^+^	277.0863	C_18_H_12_O_3_	276.08	Tanshinone Ⅰ	277.0880; 249.0931; 202.0787; 178.0785	Diterpene quinones	Danshen
67	72.56	[M + H]^+^	295.1346	C_19_H_18_O_3_	294.13	Tanshinone ⅡA	295.1322; 277.1221; 249.1272; 219.0802	Diterpene quinones	Danshen

### 3.2 Acquisition and identification of UPLC-Q-TOF-MS/MS chromatograms of the serum samples

Total ion flow diagrams of the JKZP-containing serum and blank serum samples were individually collected in positive and negative ion modes (as displayed in Supplementary Figure S1A, B). According to the multistage MS information of the samples, retention time, cleavage pattern, characterization results of the components of the original prescription, and relevant literature, a comparative analysis between JKZP-containing serum and rat blank serum samples was conducted. A total of 111 components were identified from the JKZP-containing serum, which covered 33 prototypical components and 78 metabolites. Specifically speaking, as indicated in Table 3, these mainly included 11 iridoid glycosides (all from Corni Fructus), nine phenolic acids (seven from Salviae Miltiorrhizae, one from Corni Fructus, and one from Chuanxiong Rhizoma), six oligosaccharide esters (all from Radix Polygalae), three xanthones (all from Radix Polygalae), two phenylethanoid glycosides (all from Cistanches Herba), one phthalide (from Chuanxiong Rhizoma), and one diterpene quinone (from Salviae Miltiorrhizae). Additionally, as listed in Supplementary Table S1, 78 metabolites were authenticated in the JKZP-containing serum. These metabolites were all derived from 19 compounds and, then through various metabolic pathways, such as sulfation, methylation, deglycosylation, hydroxylation, dehydroxylation, and glucuronidation, were derivatized into the 78 metabolites. The prototypical components of the JKZP-containing serum are shown in Table 3.

**TABLE 3:** Identification of prototypical components in JKZP-containing serum samples.

No.	Time (min)	Adduction	*m/z* Actual value	Molecular formula	Molecular weight	Component	MS/MS data	Classification	Source
P1	6.68	[M − H]^−^	361.078	C_14_H_18_O_11_	362.08	7-O-Galloyl-D-sedoheptulose	361.1149; 271.0470; 211.0246; 168.0063	Phenolic acids	Shanzhuyu
P2	7.05	[M − H]^−^	197.0456	C_9_H_10_O_5_	198.05	Danshensu	135.0466; 123.0462; 72.940	Phenolic acids	Danshen
P3	11.68	[M − H]^−^	345.1185	C_15_H_22_O_9_	346.13	Aucubin	345.1209; 299.1169; 89.0260	Iridoid glycosides	Shanzhuyu, Roucongrong
P4	12.07	[M − H]^−^	375.1316	C_16_H_24_O_10_	376.14	Loganic acid	375.1293; 179.0533; 169.0871	Iridoid glycosides	Shanzhuyu
P5	12.50	[M − H]^−^	461.1314	C_19_H_26_O_13_	462.14	Sibiricose A3	461.1330; 239.0604; 137.0244	Oligosaccharide esters	Yuanzhi
P6	13.04	[M − H]^-^	567.1968	C_23_H_36_O_16_	568.20	Cornusdiglycoside H	—	Iridoid glycosides	Shanzhuyu
P7	13.08	[M + FA − H]^−^	451.1434	C_17_H_26_O_11_	406.15	7α-Morroniside	405.1428; 243.0869	Iridoid glycosides	Shanzhuyu
P8	14.24	[M + FA − H]^−^	451.1458	C_17_H_26_O_11_	406.15	Morroniside	451.1475; 405.1447; 243.0890; 141.0566	Iridoid glycosides	Shanzhuyu
P9	16.31	[M + FA − H]^−^	475.1462	C_19_H_26_O_11_	430.15	Polygalatenoside A	429.1594; 307.1010; 121.0298	Oligosaccharide esters	Yuanzhi
P10	16.54	[M − H]^−^	517.1564	C_22_H_30_O_14_	518.16	Sibiricose A5	517.1464; 193.0551; 175.0399	Oligosaccharide esters	Yuanzhi
P11	18.50	[M − H]^−^	547.1705	C_23_H_32_O_15_	548.17	Sibiricose A1	547.1710; 341.1109; 205.0513	Oligosaccharide esters	Yuanzhi
P12	19.06	[M + FA − H]^−^	403.1266	C_16_H_22_O_9_	358.13	Sweroside	345.1518; 195.0650; 125.0247	Iridoid glycosides	Shanzhuyu
P13	19.26	[M + FA − H]^−^	433.1371	C_17_H_24_O_10_	388.14	Cornin	433.1481; 225.0759	Iridoid glycosides	Shanzhuyu
P14	20.89	[M + FA − H]^−^	435.1524	C_17_H_26_O_10_	390.15	Loganin	435.1544; 227.0924; 127.0393	Iridoid glycosides	Shanzhuyu
P15	22.25	[M − H]^−^	515.1164	C_25_H_24_O_12_	516.13	Isochlorogenic acid A	515.1213; 353.0885; 335.0774; 191.0568; 179.0355; 135.0454	Phenolic acids	Chuanxiong
P16	25.27	[M − H]^−^	505.1574	C_21_H_30_O_14_	506.16	Logmalicid A	505.1580; 389.1460; 227.0922	Iridoid glycosides	Shanzhuyu
P17	27.00	[M − H]^−^	505.1561	C_21_H_30_O_14_	506.16	Logmalicid B	505.1627; 227.0898	Iridoid glycosides	Shanzhuyu
P18	27.75	[M − H]^−^	537.1268	C_24_H_26_O_14_	538.13	Sibiricaxanthone A	537.1260; 387.0740; 315.0503; 285.0402; 267.0305	Xanthone	Yuanzhi
P19	28.39	[M + FA − H]^−^	607.1868	C_24_H_34_O_15_	562.19	Glomeratose A	607.2120; 561.1859; 323.0976; 237.0776	Oligosaccharide esters	Yuanzhi
P20	30.80	[M − H]^−^	785.2549	C_35_H_46_O_20_	786.26	Echinacoside	785.2515; 623.2213; 461.1679; 161.0251	Phenylethanoid glycosides	Roucongrong
P21	31.60	[M − H]^−^	567.1344	C_25_H_28_O_15_	568.14	Polygalaxanthone III	567.1385; 435.0955; 315.0490; 297.0396; 272.0289	Xanthone	Yuanzhi
P22	32.88	[M − H]^−^	567.1383	C_25_H_28_O_15_	568.14	Polygalaxanthone XI	567.1316; 345.0563; 315.0543; 272.0318	Xanthone	Yuanzhi
P23	35.01	[M − H]^−^	799.2702	C_36_H_48_O_20_	800.27	Cistanoside A	—	Phenylethanoid glycosides	Roucongrong
P32	35.61	[M − H_2_O + H]^+^	207.1024	C_12_H_16_O_4_	224.10	Senkyunolide H	207.0978; 161.0957; 133.0659; 91.0506	Phthalides	Chuanxiong
P24	38.20	[M − H]^−^	417.0819	C_20_H_18_O_10_	418.09	Salvianolic acid D	417.0830; 373.0926; 197.0454; 175.0403; 157.0303	Phenolic acids	Danshen
P25	39.75	[M − H]^−^	541.1575	C_24_H_30_O_14_	542.16	Cornuside	541.1570; 169.0145	Iridoid glycosides	Shanzhuyu
P26	40.40	[M − H]^−^	359.0788	C_18_H_16_O_8_	360.08	Rosmarinic acid	197.0453; 179.0348; 161.0247; 133.0301	Phenolic acids	Danshen
P27	41.43	[M − H]^−^	753.2277	C_34_H_42_O_19_	754.23	3,6’-Disinapoyl sucrose	753.2296; 547.1709; 519.0969; 205.0521; 190.0299	Oligosaccharide esters	Yuanzhi
P28	43.40	[M − H]^−^	537.1072	C_27_H_22_O_12_	538.11	Lithospermic acid	493.1188; 313.072; 295.0633; 185.0254	Phenolic acids	Danshen
P29	46.80	[M − H]^−^	717.1515	C_36_H_30_O_16_	718.15	Salvianolic acid B	717.1569; 519.0987; 339.0540; 321.0407	Phenolic acids	Danshen
P30	50.44	[M − H]^−^	493.1173	C_26_H_22_O_10_	494.12	Salvianolic acid A	493.1220; 313.0715; 285.0621; 185.0258	Phenolic acids	Danshen
P31	50.73	[M − H]^−^	717.1491	C_36_H_30_O_16_	718.15	Salvianolic acid Y	717.1578; 519.0947; 321.0406; 295.0653	Phenolic acids	Danshen
P33	69.88	[M + H]^+^	315.1641	C_19_H_22_O_4_	314.15	Neocryptotanshinone	253.1620; 237.0932; 223.1130; 181.1025; 165.0701	Diterpene quinones	Danshen

**FIGURE 2:**
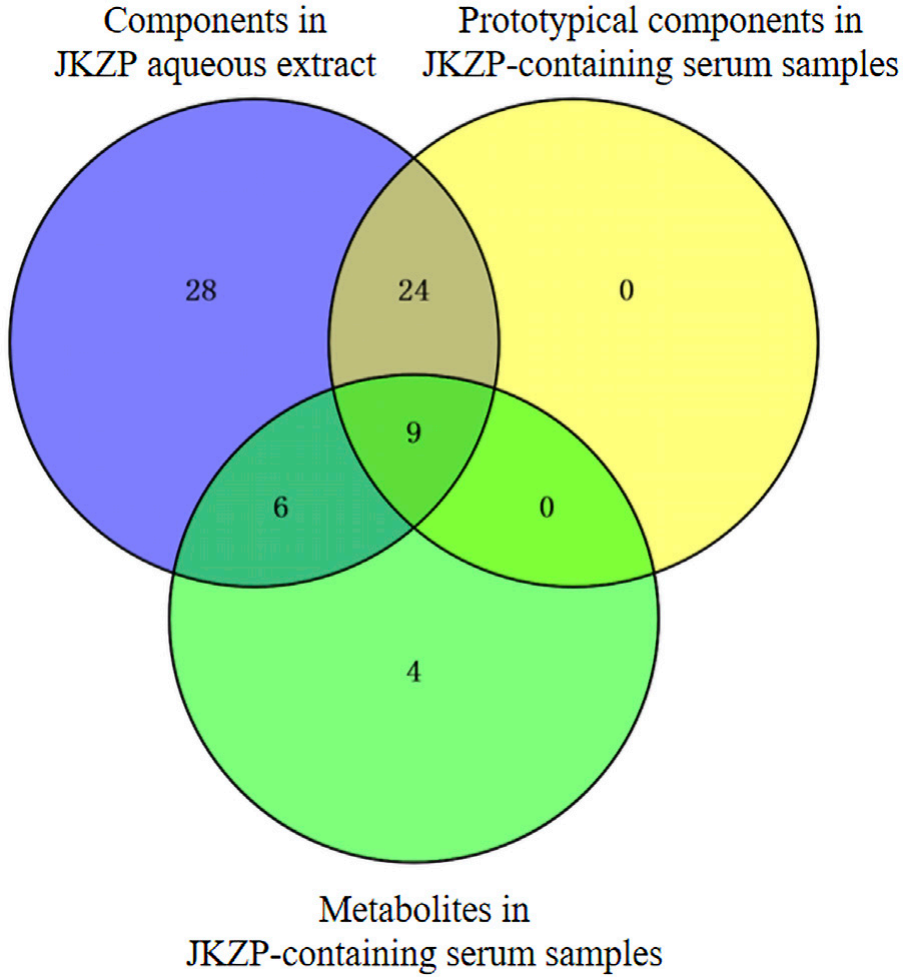
Components in the JKZP aqueous extract, prototypical components, and metabolites in JKZP-containing serum were intersected.

In the present study, all absorption into the serum corresponded to the identified components of the JKZP aqueous extract. Out of all the components, four metabolites, which were found to remain after the intersection of the metabolites with the components in the JKZP-containing serum and JKZP aqueous extract samples, could also be traced back to the components in the aqueous extract of JKZP. Specifically, pyrogallic acid was derived from gallic acid, hydroxytyrosol was derived from echinacoside, and dihydroferulic acid and caffeic acid were both derived from ferulic acid (as exhibited in Figure 2). In this study, three amino acids (L-leucine, L-tyrosine, and phenylalanine) from the tortoise plastron were identified in both the JKZP aqueous extract and serum, nevertheless, considering that the blood of a normal creature contains a variety of amino acids, such as the three aforementioned amino acids (Fernstrom et al., 1987; Wang et al., 2020b). Then, the rat blank serum was considered as the negative control to eliminate the impact of endogenously occurring substances in rats on the analysis and identification of the JKZP-containing serum. This allowed for the accurate analysis and identification of absorption into the serum of JKZP to the greatest extent possible. The intersection of the components of each sample is revealed in Figure 2.

## 4 The current status of clinical and experimental studies on JKZP

The investigations proposed that JKZP exhibited neuroprotective properties. In the study by Xu (2006), 51 patients were diagnosed with vascular dementia (VD), with 30 patients were assigned to the JKZP treatment group and 21 to the Western medicine control group. The findings revealed that the JKZP therapy group achieved a total effective rate of 76.67%, resulting in considerable enhancements in the patient's MMSE score, primary clinical symptoms, and whole blood viscosity and erythrocyte aggregation patterns. Simultaneously, JKZP also improved the cognitive impairment and self-care skills of dementia patients, demonstrating definite clinical effectiveness. JKZP could also effectively perfect the sleep quality and Chinese medical symptoms of insomnia patients with heart and kidney deficiency type, which had high effectiveness and safety (Chen et al., 2023b). Regarding the molecular mechanism, it has been verified that JKZP possessed advantageous therapeutic effects in rats suffering from focal cerebral ischemia, vascular cognitive impairment, and post-stroke depression. This encompassed safeguarding neurons against programmed cell death, stimulating the growth of cerebral angiogenesis and augmenting the reorganization of synaptic remodeling, resulting in notable enhancements in neurological impairments, cognitive impairment, and depressive behavior (Yu et al., 2013; Wu et al., 2015; Pang et al., 2016; Yu et al., 2016; Song, 2022; Zhou, 2022). However, additional investigation is necessary to elucidate the detailed pharmacological material basis for its neuroprotective impact.

## 5 Q-marker prediction analysis of neuroprotective effects exerted by JKZP

**FIGURE 3:**
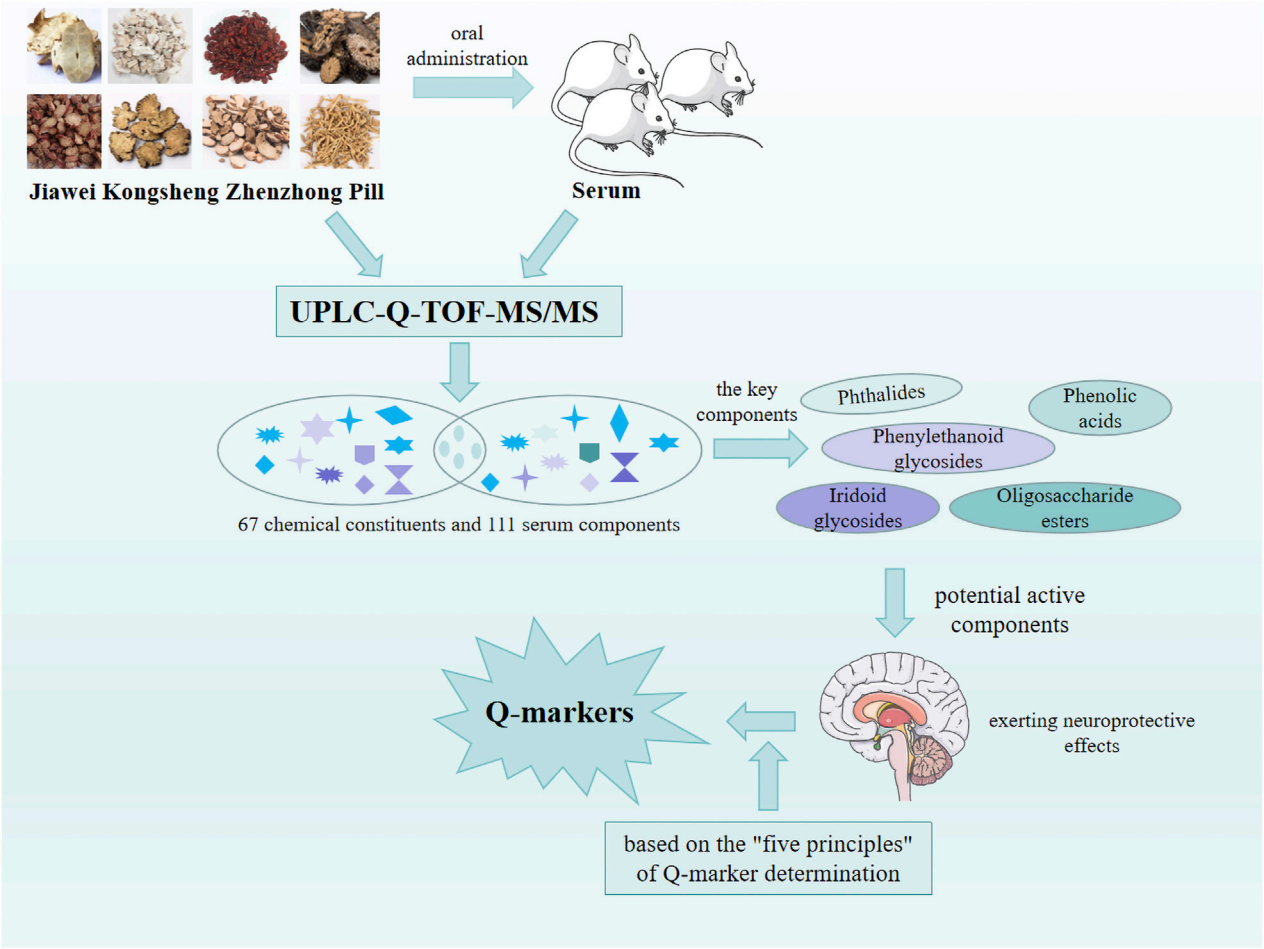
Steps in predicting Q-markers of JKZP.

Changxiao Liu, the Chinese Academy of Engineering, proposed the innovative concept of the Chinese medicine Q-marker. This concept integrated the biological attributes, manufacturing process, and prescription theory of TCM. “Quality transmission and traceability,” “compound compatibility environment,” “component specificity,” “component effectiveness,” and “component measurability” comprised the five most crucial aspects of Q-markers. These aspects effectively facilitated quality control and contributed to enhance the quality of TCM prescriptions (Liu et al., 2016). Using the UPLC-Q-TOF-MS/MS technology and “five principles” of Q-marker determination, the potential Q-markers of JKZP with neuroprotective effects were predicted. This prediction aimed to offer guidance for enhancing the overall quality control and fostering more in-depth applied research and transformative achievements for JKZP. The research strategy is demonstrated in Figure 3.

### 5.1 Q-marker prediction based on quality transmission and traceability

A comprehensive total of 67 compounds, spanning 12 categories, which includes phenolic acids, iridoid glycosides, and oligosaccharide esters, were identified from the aqueous extract of the JKZP using the UPLC-Q-TOF-MS/MS technology. With the exclusion of nucleosides and cyclic peptides, specific attributions were identified for each compound. Among them, Salviae Miltiorrhizae contributed 15 components (comprising phenolic acids and diterpene quinones); Chuanxiong Rhizoma provided nine components (primarily phenolic acids and phthalides); Acorus Tatarinowii furnished three components (phenylpropanoids and phenolic acids); Radix Polygalae yielded 14 components (mainly oligosaccharide esters and phenylpropanoids); Corni Fructus contributed 15 components (phenolic acids and iridoid glycosides); Cistanches Herba added five components (iridoid glycosides and phenylethanoid glycosides); and tortoise plastron supplied three amino acids. The study failed to identify the pertinent components of the dragon bones. According to the report, the main constituents of the dragon bones were inorganic substances, such as calcium carbonate and calcium phosphate, as well as metal elements like iron, potassium, and sodium. Consequently, the relevant components were absent from this study (Chen et al., 2023c).

In the JKZP-containing serum, a comprehensive analysis found a total of 111 blood-entry components, which comprised 33 prototypical components and 78 metabolites (as described in Table 3; Supplementary Table S1). The analysis revealed that the absorption into the serum was derived from a total of 39 original components. Specifically, Chuanxiong Rhizoma exhibited a dominance of phenolic acids and phthalides, Salviae Miltiorrhizae showcased phenolic acids and diterpene quinones, Cistanches Herba featured phenylethanoid glycosides, Corni Fructus presented iridoid glycosides, and Radix Polygalae manifested oligosaccharide esters and xanthones. Considering the aforementioned findings, it was hypothesized that phenolic acids, phthalides, phenylethanoid glycosides, iridoid glycosides, and oligosaccharide esters could potentially serve as the pivotal active components contributing to the pharmacological effects of the JKZP. All components absorbed into the serum are shown in Table 4 (after the exclusion of duplicates and endogenous components).

**TABLE 4:** All components absorbed into the serum with their classification and source.

No.	Component	Classification	Source	No.	Component	Classification	Source
1	Ferulic acid	Phenolic acids	Chuanxiong, Shichangpu	21	Cornuside	Iridoid glycosides	Shanzhuyu
2	Isochlorogenic acid A	Phenolic acids	Chuanxiong	22	Cornusdiglycoside H	Iridoid glycosides	Shanzhuyu
3	Ligustilide	Phthalides	Chuanxiong	23	Loganin	Iridoid glycosides	Shanzhuyu
4	Senkyunolide H	Phthalides	Chuanxiong	24	Loganic acid	Iridoid glycosides	Shanzhuyu
5	Senkyunolide I	Phthalides	Chuanxiong	25	Logmalicid A	Iridoid glycosides	Shanzhuyu
6	Danshensu	Phenolic acids	Danshen	26	Logmalicid B	Iridoid glycosides	Shanzhuyu
7	Gallic acid	Phenolic acids	Danshen	27	Morroniside	Iridoid glycosides	Shanzhuyu
8	Lithospermic acid	Phenolic acids	Danshen	28	Sweroside	Iridoid glycosides	Shanzhuyu
9	Protocatechuic aldehyde	Phenolic acids	Danshen	29	7-O-Galloyl-D-sedoheptulose	Phenolic acids	Shanzhuyu
10	Rosmarinic acid	Phenolic acids	Danshen	30	7α-Morroniside	Iridoid glycosides	Shanzhuyu
11	Salvianolic acid A	Phenolic acids	Danshen	31	Polygalatenoside A	Oligosaccharide esters	Yuanzhi
12	Salvianolic acid B	Phenolic acids	Danshen	32	Glomeratose A	Oligosaccharide esters	Yuanzhi
13	Salvianolic acid D	Phenolic acids	Danshen	33	Sibiricose A1	Oligosaccharide esters	Yuanzhi
14	Salvianolic acid Y	Phenolic acids	Danshen	34	Sibiricose A3	Oligosaccharide esters	Yuanzhi
15	Neocryptotanshinone	Diterpene quinones	Danshen	35	Sibiricose A5	Oligosaccharide esters	Yuanzhi
16	Tanshinone I	Diterpene quinones	Danshen	36	3,6′-Disinapoyl sucrose	Oligosaccharide esters	Yuanzhi
17	Cistanoside A	Phenylethanoid glycosides	Roucongrong	37	Polygalaxanthone III	Xanthone	Yuanzhi
18	Echinacoside	Phenylethanoid glycosides	Roucongrong	38	Polygalaxanthone XI	Xanthone	Yuanzhi
19	Aucubin	Iridoid glycosides	Shanzhuyu, Roucongrong	39	Sibiricaxanthone A	Xanthone	Yuanzhi
20	Cornin	Iridoid glycosides	Shanzhuyu				

### 5.2 Q-marker prediction based on component specificity

Chuanxiong Rhizoma is the dried rhizome of the *L. sinense* Chuanxiong plant, which belongs to the Umbelliferae family. A study conducted by Liu (2016) revealed that Chuanxiong Rhizoma had six primary pharmacologic compounds: ferulic acid, senkyunolide A/I/H, ligustilide, and levistilide A. Phthalides and phenolic acids were commonly considered to be the active components in Chuanxiong Rhizoma. Therefore, senkyunolide H and ferulic acid can be identified as the specific components of Chuanxiong Rhizoma (Liu J. et al., 2022).

Salvia miltiorrhiza is the dried root and rhizome of *S. miltiorrhiza* Bunge, which belongs to the Labiatae family. Lai et al.'s (2022) study showed that danshensu, salvianolic acid A/B, lithospermic acid, and rosmarinic acid were the main phenolic acid active components of *S. miltiorrhiza*. As for danshensu presenting with species-origin specificity, it was regarded as a unique component of *S. miltiorrhiza* (Li et al., 2018).

Cistanches Herba refers to the dehydrated succulent stem, with leaf scales, of *C. deserticola* Ma, which belongs to the Orobanchaceae family. Yang et al. (2023) identified cistanoside analogs, such as echinacoside, geniposide, cistanoside A, acteoside, and isoacteoside, as the primary active components of Cistanches Herba. Additionally, echinacoside was considered one of the distinctive constituents found exclusively in Cistanches Herba.

Corni Fructus refers to the desiccated ripe sarcocarp of *C. officinalis* Sieb. et Zucc., which belongs to the Cornaceae family. In a study conducted by Liu (2022), it was found that the primary active components of Corni Fructus were iridoid glycosides and phenolic acid chemicals, and these included gallic acid, 5-HMF, protocatechuic acid, morroniside, sweroside, loganin, and cornuside I; particularly, loganin and morroniside were the peculiar constituents in Corni Fructus (Li et al., 2017).

Radix Polygalae refers to the dehydrated root of the *Polygala tenuifolia* Willd. or *Polygala sibirica* L. plant, which belongs to the Polygalaceae family. The medical properties of this substance may be attributed to its various components, such as xanthones, saponins, oligosaccharide esters, and lipids. One specific component, known as 3,6′-disinapoyl sucrose, was particularly distinctive of Radix Polygalae (Wu et al., 2018).

### 5.3 Q-marker prediction based on the compound compatibility environment

In this prescription, JKZP comprised tortoise plastron, dragon bones, Radix Polygalae, Acorus Tatarinowii, Salviae Miltiorrhizae, Chuanxiong Rhizoma, Corni Fructus, and Cistanches Herba in the proportions of 6:6:3:3:5:4:5:4. According to the theory of TCM, tortoise plastron excelled at tonifying essence and blood, nourishing the yin (阴), submerging the yang (阳), tonifying the heart, tranquilizing the mind, and removing stagnant blood (as per “the Classic of Materia Medica”), thereby establishing its role as the sovereignty herb. Concurrently, Acorus Tatarinowii served to eliminate phlegm for resuscitation and to facilitate the movement of qi (气), promoting the alleviation of depression. Dragon bones contributed to tranquillization with a potent prescription, while Chuanxiong Rhizoma was utilized for activating blood circulation, eliminating blood stasis, and facilitating the movement of qi (气), thereby promoting relief from depression. The trio of herbs functioned as ministerial herbs. Then, Cistanches Herba benefited essence and blood and tonified kidney yang; Salviae Miltiorrhizae activated blood circulation and eliminated blood stasis; Radix Polygalae stabilized the mind and strengthened the intellect; Corni Fructus consolidated yin(阴) and replenished essence. They also served as assistant herbs. Subsequent to this, Chuanxiong Rhizoma, proficient in clearing the way and ascending toward the head and eyes, was employed to guide the medicines to the site of illness, functioning as a courier herb. Overall, the combination of these herbs aimed to tonify the kidneys, benefit the essence, dissipate phlegm, eliminate blood stasis, tranquilize the mind, and alleviate depression.

Chinese medicines are predominantly utilized in clinical practice through the form of prescriptions. Contemporary pharmacological research has demonstrated that varied combinations or dosages of herbs would result in variations in the effectiveness and underlying pharmacodynamic properties. Hence, it is imperative to anticipate the distinctive quality indicators pertaining to the neuroprotective properties of JKZP based on the TCM prescriptions principles. The research conducted by Liu M. et al. (2022) has demonstrated a significant increase in the levels of danshensu sodium, protocatechuic aldehyde, rosmarinic acid, salvianolic acid A/B, cryptotanshinone, tanshinone Ⅰ, and tanshinone ⅡA in the extract of Salviae Miltiorrhizae and Chuanxiong Rhizoma when the two were combined. The combination of Chuanxiong Rhizoma and Salviae Miltiorrhizae with *Pueraria lobata* (Willd.) Ohwi for treating cardiac and cerebral diseases resulted in the presence of soy sapogenins, genistein, 3′-methoxy soy sapogenins, formononetin, and cryptotanshinone in the bloodstream. These compounds were closely associated with the targets AKR1B1, CA2, CA1, and ALDH2 (Tang Y. et al., 2022). It was found that the combination of Corni Fructus and *Rehmannia glutinosa* (Gaertn.) resulted in a notable increase in the dissolution of loganin, a bioactive compound found in Corni Fructus (Zhou and Cong, 2018). Lv et al. (2016) conducted a comprehensive analysis of the chemical components of Radix Polygalae before and after pairing it with Acorus Tatarinowii. The study found that the levels of the eight chemical constituents, such as tenuifoliside, sibiricose A5, and 3,6′-disinapoyl sucrose, remained unchanged. However, the concentration of the volatile constituent cis-β-asarone significantly decreased (Zhang et al., 2015). The role and therapeutic effect of Chinese herbal prescriptions could be attributed to the synergy between the individual drugs and active compounds. It was observed that when the same single drugs were combined in a compound environment, they exhibited different pharmacological material bases and mechanisms of action, resulting in varied therapeutic effects.

### 5.4 Q-marker prediction based on the association between components and effectiveness

The properties of the components determined the pharmacological effects, constituting the core element of the Q-marker, and were pivotal for the quality control in prescriptions. Synthesizing the above theories and analytical results, JKZP exhibited neuroprotective effects potentially related to the key absorption into the serum from Chuanxiong Rhizoma, Salviae Miltiorrhizae, Cistanches Herba, Corni Fructus, and Radix Polygalae.

#### 5.4.1 Chuanxiong Rhizoma

The compounds of Chuanxiong Rhizoma, as recorded in the “Chinese Materia Medica,” encompassed senkyunolide, ferulic acid, and caffeic acid. Upon oral ingestion, the medicines entered the body and exerted their therapeutic effects either directly as basic components or after undergoing a series of metabolic processes. In the study by Liu Z. et al. (2022), rats were orally administered senkyunolide H at a dosage of 10 mg kg^−1^. After 24 h, the plasma, urine, bile, and feces samples were collected for analysis. A total of 32 metabolites were detected, with the primary metabolic reactions involving oxidation, hydrogenation, methylation, acetylation, dehydroxylation, glucuronidation, esterification, and cysteine binding.

Studies have indicated that senkyunolide H exhibited neuroprotective effects in animals with ischemia–reperfusion injury or by safeguarding neuronal cells from injury induced by oxygen–glucose deprivation and reperfusion (OGD/R) via the cAMP-PI3K/AKT signaling pathway or the PI3K/AKT/NF-κB signaling pathway. Additionally, senkyunolide H decreased the release of inflammatory factors in the brain tissues of mice with middle cerebral artery occlusion and enhanced the ability of neurons to resist apoptosis, leading to a decrease in neurological impairment, volume of brain tissue damage due to the lack of blood supply, and mortality of neurons, thereby demonstrating notable neuroprotective effects (Zhang et al., 2019). It bolstered the ability of neurons to withstand oxidative stress by reducing the generation of reactive oxygen species, mitigating the loss of mitochondrial membrane potential, restricting the release of cytochrome C, and decreasing the levels of malondialdehyde. Simultaneously, it augmented antioxidant enzyme activities, such as superoxide dismutase, catalase, and glutathione peroxidase (Luo et al., 2019). It also attenuated neuroinflammation by blocking the Prx1/TLR4/NF-kB, ERK and NF-κB signaling pathways (Han et al., 2018; Tan et al., 2022). These combined mechanisms contribute to its neuroprotective effects. Thus, it was further anticipated that senkyunolide H might function as a quality indicator for JKZP.

#### 5.4.2 Salviae Miltiorrhizae

The primary active substances of Salviae Miltiorrhizae are phenolic acids and diterpene quinones (Xu et al., 2018). Danshensu and tanshinone ⅡA serve as the quality control indicators for the antioxidant and anti-apoptotic properties of Salviae Miltiorrhizae aqueous extracts, respectively (Zhou et al., 2012). The study conducted by Lai et al. (2022) analyzed the levels of six phenolic acids in a digested extract of *S. miltiorrhiza* using an artificial gastric fluid. The investigated components were danshensu, lithospermic acid, and salvianolic acid A/B, whose bioaccessibility followed the following order, from the highest to lowest: the percentages of danshensu (50.19%), salvianolic acid B (33.44%), lithospermic acid (27.34%), salvianolic acid A (21.71%), and rosmarinic acid (12.31%), respectively. A higher bioaccessibility indicated that the components could be readily metabolized and assimilated by the stomach and intestines, enabling them to efficiently deliver their therapeutic effect. Evidence has suggested that phenolic acids are more readily digested and released by artificial gastric juice. The experimental findings of Hu (2015) proved that when rats were administered phenolic acids intravenously via the tail vein, the presence and peak concentration of danshensu in rat plasma were significantly higher than those of salvianolic acid B, and the mean residence time and half-life of danshensu were significantly longer.

Numerous studies have demonstrated the significant effectiveness of danshensu in various aspects, which include its ability to restrain oxidative stress (Wang et al., 2020a), decrease neuroinflammation (Han et al., 2019; Ye et al., 2020; Bai et al., 2023), prevent apoptosis (Guo et al., 2015; Fan et al., 2016), promote angiogenesis (Yin et al., 2017), ameliorate neurogenesis (Wei et al., 2018), improve the mitochondrial function (Xue et al., 2022), and alleviate the toxic effects of Aβ proteins on the brain (Zheng et al., 2023). These findings suggest its potential therapeutic applications in neurodegenerative disorders (such as Parkinson's disease and Alzheimer's disease), cerebral ischemia or ischemia/perfusion injury, and other diseases related to the nervous system. Consequently, danshensu could be anticipated as one of the indicators of quality for JKZP.

#### 5.4.3 Cistanches Herba

Cistanches Herba has been identified with more than 150 compounds (Song et al., 2021), and the “Pharmacopoeia of the People's Republic of China” has recorded echinacoside as its key active ingredient. In the study by Yan (2018), a thorough analysis was conducted on the plasma, urine, and feces of rats that were given Cistanches Herba extracts orally. The study found a total of 82 characteristic compounds, with echinacoside being one of the main components.

Previous studies have indicated that echinacoside exerted a wide range of neuroprotective effects, such as anti-neuroinflammation (Zhang et al., 2017a; Gao et al., 2020; Lu et al., 2022; Yang et al., 2022), promotion of hippocampal neurogenesis (Lu et al., 2022), inhibition of glutamatergic excitotoxicity (Lu et al., 2016), reduction of oxidative stress (Zhao et al., 2016; Zheng et al., 2019), prevention of apoptosis (Zhu et al., 2013; Wei et al., 2019), enhancement of mitochondrial function (Ma et al., 2019), mitigation of β-amyloid neurotoxicity (Shiao et al., 2017), stimulation of autophagy (Chen et al., 2019), and suppression of endoplasmic reticulum stress (Zhang et al., 2017b). These effects have contributed to improving learning memory and cognitive disorders, as well as depressive behaviors. Consequently, echinacoside has extensive applications in neurological-related diseases.

#### 5.4.4 Corni Fructus

Iridoid glycosides were the characteristic components of Corni Fructus, with 91 compounds of this class being isolated. The “Pharmacopoeia of the People's Republic of China” designated the total content of morroniside and loganin as a quality control index for Corni Fructus (Fan et al., 2020). A study conducted by Li (2007) examined the pharmacokinetic process of loganin in rats following the oral administration of a single dosage of loganin and Corni Fructus extract. Administering loganin (20 mg kg^−1^) and Corni Fructus extract (40 mg kg^−1^) orally to rats led to a notable elevation in their blood concentration. The blood concentration of loganin reached its highest level at 69 min, whereas that of the Corni Fructus extract peaked at 51 min. The compounds exhibited elimination half-lives of 93.6 min and 99.4 min, respectively.

Loganin exhibited anti-neuroinflammatory properties by boosting the polarization of M2 microglia, lowering the release of inflammation-related mediators, and playing a protective role in ischemic stroke mouse models (Huo et al., 2023). Loganin has also demonstrated effects such as anti-neuronal apoptosis (Kwon et al., 2011; Tseng et al., 2019), anti-oxidative stress (Kwon et al., 2011), improvement of mitochondrial function (Zhou et al., 2023), modulation of neurotransmitter release (Shi et al., 2019), reduction in neuronal damage, and alleviation of cognitive deficits in animal models. It shows potential for treating ischemic stroke and neurological illnesses.

#### 5.4.5 Radix Polygalae

Modern pharmacological studies have indicated that extracts from Radix Polygalae attenuated neuronal cell damage and ameliorated cognitive impairments associated with learning and memory in various animal models of neurodegenerative disorders (Yuan et al., 2021). The compound 3,6′-disinapoyl sucrose was included in the “Pharmacopoeia of the People's Republic of China” as a quality control marker for Radix Polygalae. In the study by Xiong et al. (2023), rats were orally administered the Radix Polygalae extract at a dosage of 5 g kg^−1^. The UPLC-MS/MS approach was employed to ascertain the compounds present in the plasma. The findings indicated that the plasma blood concentration of 3,6′-disinapoyl sucrose peaked at 2 h, reaching a maximum concentration of 241.70 ± 15.18 ug·L^−1^.

The compound 3,6′-disinapoyl sucrose possesses the properties of anti-oxidative stress (Shi et al., 2015; Tang X. et al., 2022), counteracting glutamate excitotoxicity (Hu et al., 2014), enhancing neuroplasticity (Hu et al., 2010), and promoting neurogenesis (Wang et al., 2021). Therefore, it was frequently employed to treat conditions such as ischemic stroke, insomnia, amnesia, and depressive disorders.

### 5.5 Q-marker prediction based on component measurability

The composition of TCM prescriptions is intricate and diverse. Clarifying their key active components is crucial for elucidating the mechanism of their efficacy. Therefore, the Q-marker should be measurable. Studies by Qu et al. (2020) and Li et al. (2008) have detected danshensu in the plasma of rats gavaged with the Salviae Miltiorrhizae aqueous extract. The concentration of danshensu in the plasma ranged from 5 to 500 ng mL^−1^, demonstrating a linear relationship with its transformation. The study by Zhao et al. (2020) used the UHPLC-MS/MS technology to assess the pharmacokinetics and bioavailability of active components of Radix Polygalae in rat serum. The results have shown that the absolute bioavailability of sibiricose A5, A6, and 3,6′-disinapoyl sucrose was 3.25%, 2.95%, and 2.36%, respectively. The study by Yang et al. (2020) used the HPLC method to quantify the components of Cistanches Herba, such as echinacoside, cistanoside A, acteoside, and isoacteoside. This approach facilitated a comprehensive evaluation of the quality of the prepared slices. The study by Liu (2022) employed a traditional, reliable, and stable liquid-phase method to determine the quality of Corni Fructus, which included morroniside, sweroside, loganin, gallic acid, 5-HMF, protocatechuic acid, and cornuside I. The biological activity results indicated that these seven chemical ingredients could be used as Q-markers for evaluating the quality of Corni Fructus. Yalu et al.'s (2023) research identified the main components of Chuanxiong Rhizoma by liquid chromatography, encompassing senkyunolide H, chlorogenic acid, n-butylphenol, ligustrazine, ferulic acid, ligustilide, and others.

**FIGURE 4:**
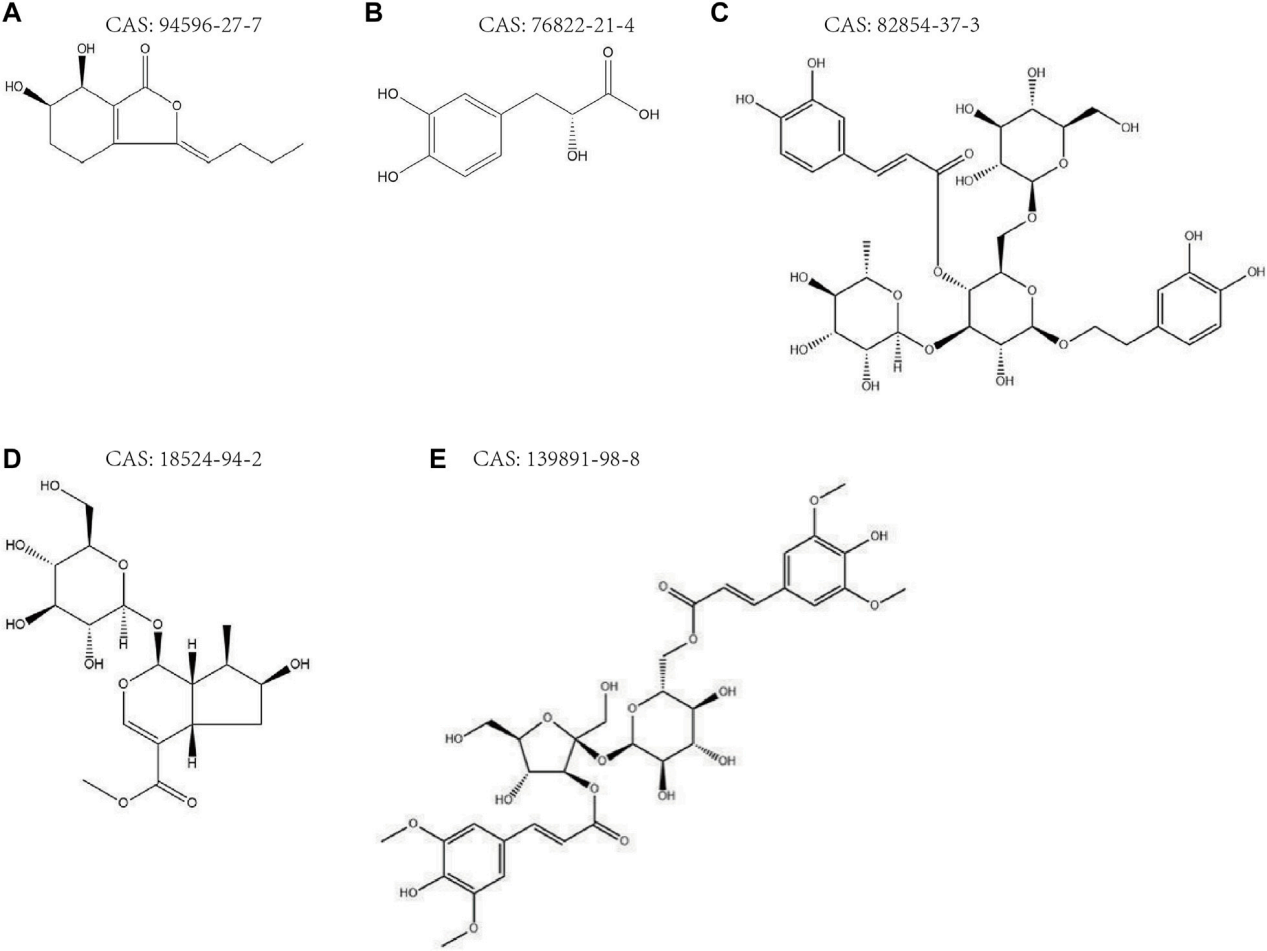
**(A–E)** Chemical structures of JKZP Q-markers. **(A)** Senkyunolide H; **(B)** danshensu; **(C)** echinacoside; **(D)** loganic acid; **(E)** 3,6′-disinapoyl sucrose.

In conclusion, senkyunolide H, danshensu, echinacoside, loganic acid, and 3,6′-disinapoyl sucrose, with high proprietary and measurability, were predicted to be the key pharmacological bases for the neuroprotective effects of JKZP based on the “five principles” of Q-marker determination. The chemical structures of the Q-marker of JKZP are shown in Figure 4 , the maps of peaks in the JKZP aqueous extract and JKZP-containing serum are revealed in Supplementary Figures S2, S3, and the mechanisms of neuroprotection are listed in Table 5.

**TABLE 5:** Q-markers of JKZP and its neuroprotective mechanisms.

No.	Compound	Source	Disease/Model	Effect	Mechanism	Reference
1	Senkyunolide H	Chuanxiong	Ischemic stroke model *in vivo*	Upregulating the level of cAMP, p-CREB, p-AKT, p-PDK1, and PKA	Activating cAMP-PI3K/AKT signaling pathway	Jiang et al. (2022)
2	Senkyunolide H	Chuanxiong	Ischemic stroke model *in vitro* and *in vivo*	Anti-inflammation and anti-apoptosis	Activating the PI3K/Akt/NF-κB pathway	Zhang et al. (2019)
3	Senkyunolide H	Chuanxiong	PC12 cells induced oxidative stress by 1-methyl-4-phenylpyridinium *in vitro*	Anti-oxidative and anti-apoptosis	Inhibiting the NF-κB/JNK/MAPK pathway	Luo et al. (2019)
4	Senkyunolide H	Chuanxiong	Intracerebral hemorrhage model *in vivo*	Anti-inflammation	Inhibiting the Prx1/TLR4/NF-κB pathway	Han et al. (2018)
5	Senkyunolide H	Chuanxiong	Lipopolysaccharide-mediated neuroinflammation and oxidative stress in BV2 microglia cells *in vitro*	Anti-oxidative and anti-inflammation	Inhibiting the ERK and NF-κB pathways	Tan et al. (2022)
6	Danshensu	Danshen	Parkinson's disease models *in vitro* and *in vivo*	Anti-oxidative	Activating the PI3K/AKT/Nrf2 pathway	Wang et al. (2020b)
7	Danshensu	Danshen	Bone marrow–derived macrophages activated by a Toll-like receptor 2 (TLR2) agonist Pam3CSK4	Anti-inflammation	Inhibiting the NF-κB signaling pathway	Ye et al. (2020)
8	Danshensu	Danshen	Astrocytes and microglia with cerebral ischemia–reperfusion injury *in vitro* and *in vivo*	anti-inflammation	Polarizing astrocytes from A1 to A2 and microglia from M1 to M2	Bai et al. (2023)
9	Danshensu	Danshen	Parkinson's disease models *in vivo* induced by 1-methyl-4-phenyl-1.2,3,6-tetrahydropyridine (MPTP)	Anti-inflammation	Inhibiting Iba1-positive cells in the substantia nigra and reducing the levels of interleukin-1β and tumor necrosis factor-α in the striatum	Han et al. (2019)
10	Danshensu	Danshen	Ischemic stroke model *in vivo*	Anti-autophagy and anti-apoptosis	Activating the mTOR pathway	Fan et al. (2016)
11	Danshensu	Danshen	Ischemic stroke model *in vivo*	Anti-apoptosis	Activating the PI3K/Akt signal pathway	Guo et al. (2015)
12	Danshensu	Danshen	Myocardial infarction *in vivo*	Accelerating angiogenesis	Activating SDF-1/CXCR4 axis	Yin et al. (2017)
13	Danshensu	Danshen	Focal cerebral ischemia in mice *in vivo*	Enhancing neurogenesis	Increasing the newly formed arteries and the diameter of collateral arteries, leading to enhanced local cerebral blood flow recovery after a stroke	Wei et al. (2018)
14	Danshensu	Danshen	Platelet activation by analyzing aggregation and adhesion *in vitro*	Preventing mitochondrial dysfunction and inhibiting platelet activation	Activating the SIRT1/ROS/mtDNA pathway	Xue et al. (2022)
15	Danshensu	Danshen	Aβ(1–42) fibrillation and neuron-like SH-SY5Y cells *in vitro*	Anti-neurotoxicity	Inhibiting Aβ(1–42) aggregation and associated proteinopathies through regulation of the apoptotic pathway	Zheng et al. (2023)
16	Echinacoside	Roucongrong	Parkinson's disease models *in vivo* with 1-methyl-4-phenyl-1.2,3,6-tetrahydropyridine-induced damage	Anti-inflammation	Inhibiting the p38 MAPK and NF-κB p52 signals	Zhang et al. (2017a)
17	Echinacoside	Roucongrong	Parkinson's disease models *in vivo* with 1-methyl-4-phenyl-1.2,3,6-tetrahydropyridine-induced damage	Anti-inflammation	Inhibiting the NLRP3/caspase-1/IL-1β signaling pathway	Gao et al. (2020)
18	Echinacoside	Roucongrong	Parkinson's disease models *in vivo* and LPS-induced BV2 cells *in vitro*	Anti-inflammation	Inhibiting the IL-6/JAK2/STAT3 pathway	Yang et al. (2022)
19	Echinacoside	Roucongrong	Depression mice and N9 microglial cells stimulated by LPS	Anti-inflammation and improving hippocampal neurogenesis	Activating CREB/BDNF and JAK1/STAT3 signaling pathway	Lu et al. (2022)
20	Echinacoside	Roucongrong	4-Aminopyridine-evoked glutamate release in rat cerebrocortical nerve terminals	Reducing glutamate-induced toxicity	Reducing voltage-dependent Ca (2+) entry and subsequent suppression of protein kinase C activity	Lu et al. (2016)
21	Echinacoside	Roucongrong	Hypobaric hypoxia-induced memory impairment in C57 mice *in vivo*	Anti-oxidative	Activating the Keap1/Nrf2/ARE signaling pathway	Zheng et al. (2019)
22	Echinacoside	Roucongrong	Hypoxic–ischemic brain damage model *in vivo*	Anti-oxidative and anti-apoptosis	Recovering the antioxidant enzyme activities decreasing the caspase-3 levels and increasing the Bcl-2/Bax ratio	Wei et al. (2019)
23	Echinacoside	Roucongrong	Parkinson's disease model *in vitro* and *in vivo*	Anti-apoptosis	Inhibiting the ROS/ATF3/CHOP pathway	Zhao et al. (2016)
24	Echinacoside	Roucongrong	Neuronal cells and non-neuronal cells with rotenone injury *in vitro*	Anti-apoptosis	Activating Trk receptors and their downstream signal pathways	Zhu et al. (2013)
25	Echinacoside	Roucongrong	SH-SY5Y cells treated by an inhibitor of complexes I–IV	Improving mitochondrial dysfunction	Enhancing complex II activity and mitochondrial respiration	Ma et al. (2019)
26	Echinacoside	Roucongrong	Amyloid β peptide 1–42 [(Aβ(1–42)]–treated SH-SY5Y cells and an Aβ(1–42)-infused rat *in vitro* and *in vivo*	Anti-neurotoxicity	Blocking amyloid deposition via inhibiting amyloid oligomerization and reversing the cortical cholinergic neuronal function via decreasing amyloid neurotoxicity	Shiao et al. (2017)
27	Echinacoside	Roucongrong	Parkinson's disease model *in vitro* and *in vivo* with MPTP/MPP-induced neurotoxicity	Enhancing autophagy in neurons	Binding to SIRT1 directly and affecting FoxO expression	Chen et al. (2019)
28	Echinacoside	Roucongrong	Endoplasmic reticulum stress model of rats and PC12 cells treated with 6-hydroxydopamine *in vitro* and *in vivo*	Anti-endoplasmic reticulum stress	Inhibiting the Grp94/Bip/ATF4/CHOP pathway	Zhang et al. (2017b)
29	Loganin	Shanzhuyu	Ischemic stroke model *in vivo* and LPS-stimulated BV2 cells *in vitro*	Enhancing autophagy and anti-inflammation	Regulating α7nAChR-mediated microglial polarization	Huo et al. (2023)
30	Loganin	Shanzhuyu	SH-SY5Y cells–induced neuronal toxicity by H_2_O_2_	Anti-apoptosis	Inhibiting JNK/p38 and ERK 1/2 MAPKs	Kwon et al. (2011)
31	Loganin	Shanzhuyu	Primary mesencephalic neuronal cells treated with 1-methyl-4-phenylpyridinium *in vitro*	Anti-apoptosis	Enhancing neurotrophic signaling, activating IGF-1R/GLP-1R, and inhibiting the RhoA/ROCK pathway	Tseng et al. (2019)
32	Loganin	Shanzhuyu	Alzheimer's disease models *in vitro* and *in vivo*	Promoting mitophagy and mitochondrial function	Activating cell mitophagy	Zhou et al. (2023)
33	Loganin	Shanzhuyu	Insomnia models *in vivo*	Regulating neurotransmitter release	Modification of the serotonergic system and GABAergic neurons	Shi et al. (2019)
34	3,6′-Disinapoyl sucrose	Yuanzhi	Aβ(1–42)-induced neurotoxicity in *Caenorhabditis elegans*	Anti-oxidative	Regulating expression of genes related to antioxidation and autophagy	Tang et al. (2022b)
35	3,6′-Disinapoyl sucrose	Yuanzhi	SH-SY5Y cells induced by glutamate and H_2_O_2_ *in vitro*	Reducing glutamate and H_2_O_2_-induced toxicity	Activating the CaMKII and ERK1/2 pathway	Hu et al. (2014)
36	3,6′-Disinapoyl sucrose	Yuanzhi	Chronic mild stress rats *in vivo*	Improving levels of CAM-L1, laminin, and BDNF	Enhancing hippocampal neuronal plasticity	Hu et al. (2010)
37	3,6′-Disinapoyl sucrose	Yuanzhi	Alzheimer's disease model *in vivo*	Increasing hippocampal neurogenesis	Strengthening neural stem cell proliferation and neuronal differentiation	Wang et al. (2021)

## 6 Conclusion

JKZP exhibited the characteristics of tonifying the kidneys and marrow, promoting blood circulation, resolving stasis, and alleviating depression. Consequently, it has been utilized to address conditions such as stroke, post-stroke sequelae, vascular cognitive impairment, or dementia associated with kidney essence insufficiency, blood stasis obstruction, and blockage syndrome. It has had a notable impact on neuroprotection by inhibiting neuronal apoptosis, stimulating angiogenesis, and enhancing synaptic remodeling. Nevertheless, the lack of a distinct material foundation has severely hindered its progress and utilization. Hence, the article employed the UPLC-Q-TOF-MS/MS technology to examine and identify the compounds in the JKZP aqueous extract and JKZP-containing serum samples. This can serve as a source of information to further elucidate the pharmacological substance basis of JKZP.

The current study marked the first instance of analyzing and identifying the compounds of the aqueous extract of JKZP, along with the absorption into the serum. The aqueous extract contained a total of 12 chemical categories and 67 unique components identified. The JKZP-containing serum encompassed 111 components, comprising 33 prototype components and 78 metabolites. These components were derived from a pool of 39 original components. Iridoid glycosides, phenolic acids, oligosaccharide esters, phenylethanoid glycosides, and phthalides were posited as the potential key pharmacophore basis of JKZP. A comparison of the obtained compounds with the literature demonstrated the satisfactory results of this assay, with key compounds detected in all herbs except for dragon bones. The neuroprotective effects of JKZP were attributed to five specific components: senkyunolide H, danshensu, echinacoside, loganin, and 3,6 ′ -disinapoyl sucrose. These components have strong measurability and characterization and are believed to be the main pharmacological foundations for the neuroprotective effects of JKZP, according to the “five principles” of Q-marker determination. Prior studies have emphasized the function of the blood–brain barrier (BBB) in safeguarding the brain from exogenous, neurotoxic, and other substances in the blood, thereby impeding the passage of nearly 98% of small-molecule drugs. Therefore, the ability to traverse the BBB and target particular regions of the brain tissue is an essential property of chemicals or molecules that exhibit neuroprotective effects (Hornok et al., 2022; Katila et al., 2022). As previously stated, there is compelling evidence that all the aforementioned predicted Q-markers described could cross the BBB and provide varying degrees of neuroprotection (Li, 2007; Zhang et al., 2011; Wang et al., 2013; Zhu et al., 2013; Yang, 2019). Thus, they might be regarded as the Q-marker for JKZP.

Ultimately, this work primarily examined the properties of JKZP compounds and their uptake into the rat serum through the utilization of UPLC-Q-TOF-MS/MS technology. Senkyunolide H, danshensu, echinacoside, loganin, and 3,6′-disinapoyl sucrose have been predicted as Q-markers for JKZP. These findings provide empirical evidence to support the assessment of the quality and application of JKZP, serving as a reliable foundation for the theoretical advancement of prescriptions. However, certain constraints endured, Acorus Tatarinowii and Chuanxiong Rhizoma in this prescription contained more volatile oil components, which brought out the effects of opening the mind, awakening the brain, and alleviating depression. The study did not specifically prioritize the detection and identification of these volatile oil components nor did it reconfirm the neuroprotective benefits of Q-markers on neurodegenerative and ischemic stroke illnesses. Subsequent studies may concentrate on a comprehensive examination of volatile oil components and perform trials both *in vivo* and *in vitro* to validate the neuroprotective benefits of JKZP's Q-markers with the aim to further enhance the fundamental research on the pharmacodynamic substances of JKZP.

## Data Availability

The original contributions presented in the study are included in the article/Supplementary Material; further inquiries can be directed to the corresponding authors.
