# Supplementary material for: Jiawei Kongsheng Zhenzhong Pill: marker compounds, absorption into the serum (rat), and Q-markers identified by UPLC-Q-TOF-MS/MS
Source: Front Pharmacol. 2024 Feb 5;15:1328632. doi: 10.3389/fphar.2024.1328632 (PMC10875140; doi:10.3389/fphar.2024.1328632)
Supplement: Supplementary file 1 [file Table1.docx]

Supplementary Material

## Supplementary Tables

**Supplementary Table S1.** Identification of metabolites in JKZP-containing serum samples

| **No.** | **Time（min）** | **Adduction** | ***m/z* Actual value** | **Molecular formula** | **Molecular weight** | **Ingredients and metabolic pathways** | **MS/MS data** |
| --- | --- | --- | --- | --- | --- | --- | --- |
| M1 | 6.04 | [M-H]^-^ | 204.9815 | C_6_H_6_O_6_S | 205.99 | Pyrogallic acid+sulfation | 204.9815;125.0246;69.0346 |
| M2 | 6.79 | [M-H]^-^ | 233.0125 | C_8_H_10_O_6_S | 234.02 | Hydroxytyrosol+sulfation | 233.0124;153.0554;138.0320;120.0220 |
| M3 | 7.06 | [M-H]^-^ | 247.0291 | C_9_H_12_O_6_S | 248.04 | Hydroxytyrosol+methylation+sulfation | 247.0279;167.0707;93.0369 |
| M4 | 7.50 | [M-H]^-^ | 233.0135 | C_8_H_10_O_6_S | 234.02 | Hydroxytyrosol+sulfation | 233.0092;153.0558;123.0450 |
| M5 | 8.12 | [M-H]^-^ | 233.0116 | C_8_H_10_O_6_S | 234.02 | Hydroxytyrosol+sulfation | 233.0133;153.0566;135.0451;123.0460 |
| M6 | 8.84 | [M-H]^-^ | 313.0577 | C_13_H_14_O_9_ | 314.06 | Protocatechuic aldehyde+glucuronidationn | / |
| M7 | 9.07 | [M-H]^-^ | 307.0502 | C_11_H_16_O_8_S | 308.06 | Loganin+deglycosylation+sulfation | 307.0492;227.0952;209.0839;194.0597;179.0353 |
| M8 | 9.21 | [M-H]^-^ | 181.0510 | C_9_H_10_O_4_ | 182.06 | Danshensu+Dehydroxylation | / |
| M9 | 9.29 | [M-H]^-^ | 262.9877 | C_8_H_8_O_8_S | 263.99 | Gallic acid+methylation+sulfation | 262.9947;183.0277;168.0051 |
| M10 | 9.63 | [M-H]^-^ | 307.0491 | C_11_H_16_O_8_S | 308.06 | Loganin+deglycosylation+sulfation | 307.0478;227.0922;212.0693;197.0459;153.0197 |
| M11 | 9.65 | [M-H]^-^ | 371.0978 | C_16_H_20_O_10_ | 372.11 | Ferulicacid+hydrogenation+glucuronidation | 371.1007;195.0655;180.0437;133.0666 |
| M12 | 9.83 | [M-H]^-^ | 247.0295 | C_9_H_12_O_6_S | 248.04 | Hydroxytyrosol+methylation+sulfation | 247.0260;167.0706;152.0677 |
| M13 | 10.06 | [M-H]^-^ | 261.0088 | C_9_H_10_O_7_S | 262.01 | Danshensu+Dehydroxylation +sulfation | / |
| M14 | 10.44 | [M-H]^-^ | 261.0083 | C_9_H_10_O_7_S | 262.01 | Danshensu+Dehydroxylation +sulfation | 261.0057;181.0506;137.0607 |
| M15 | 11.03 | [M-H]^-^ | 247.0288 | C_9_H_12_O_6_S | 248.04 | Hydroxytyrosol+methylation+sulfation | 247.0226;167.0747;152.0495;137.0598 |
| M16 | 11.08 | [M-H]^-^ | 421.1348 | C_17_H_26_O_12_ | 422.14 | Morroniside+hydroxylation | 259.1300;165.0159;128.0350 |
| M17 | 11.38 | [M-H]^-^ | 239.0576 | C_11_H_12_O_6_ | 240.06 | Danshensu+Acetylation | / |
| M18 | 11.50 | [M-H]^-^ | 391.1248 | C_16_H_24_O_11_ | 392.13 | Morroniside+demethylation | / |
| M19 | 11.50 | [M-H]^-^ | 247.0284 | C_9_H_12_O_6_S | 248.04 | Hydroxytyrosol+methylation+sulfation | 247.0294;167.0703;123.0458 |
| M20 | 11.51 | [M-H]^-^ | 275.0226 | C_10_H_12_O_7_S | 276.03 | Dihydroferulic acid+sulfation | 275.0247;195.0673;180.0439 |
| M21 | 11.65 | [M-H]^-^ | 307.0502 | C_11_H_16_O_8_S | 308.06 | Loganin+deglycosylation+sulfation | 307.0466;227.9919;212.0678;197.0446;153.0186 |
| M22 | 12.17 | [M-H]^-^ | 275.0234 | C_10_H_12_O_7_S | 276.03 | Dihydroferulic acid+sulfation | 275.0199;195.0666;180.0430 |
| M23 | 12.50 | [M-H]^-^ | 258.9919 | C_9_H_8_O_7_S | 260.00 | Caffeic acid+sulfation | 179.0348;164.0482;135.0453 |
| M24 | 12.52 | [M-H]^-^ | 307.0482 | C_11_H_16_O_8_S | 308.06 | Loganin+deglycosylation+sulfation | 307.0512;227.0906;194.0580;179.0345 |
| M25 | 12.65 | [M-H]^-^ | 245.0132 | C_9_H_10_O_6_S | 246.02 | Danshensu+didehydroxylation+sulfation | / |
| M26 | 12.78 | [M-H]^-^ | 355.0695 | C_15_H_16_O_10_ | 356.07 | Caffeic acid+glucuronidation | 179.0341;158.0815;135.0445 |
| M27 | 12.87 | [M-H]^-^ | 403.1257 | C_17_H_24_O_11_ | 404.13 | Morroniside+deglycosylation+dehydration+hydrogenation+glucuronidation | 403.1251;212.0678;197.0461;158.0825 |
| M28 | 12.94 | [M-H]^-^ | 275.0242 | C_10_H_12_O_7_S | 276.03 | Dihydroferulic acid+sulfation | 275.0237;195.0675;136.0531 |
| M29 | 13.17 | [M-H]^-^ | 273.0073 | C_10_H_10_O_7_S | 274.01 | Ferulic acid+sulfation | 193.0504;178.0256;134.0366 |
| M30 | 13.84 | [M-H]^-^ | 355.0682 | C_15_H_16_O_10_ | 356.07 | Caffeic acid+glucuronidation | 355.0634;234.9152;179.0332;135.0447 |
| M31 | 14.80 | [M-H]^-^ | 273.0090 | C_10_H_10_O_7_S | 274.01 | Ferulic acid+sulfation | 273.0077;193.0510;178.0280;134.0382 |
| M32 | 15.41 | [M-H]^-^ | 307.0491 | C_11_H_16_O_8_S | 308.06 | Loganin+deglycosylation+sulfation | 307.0483;227.0914;127.0406;101.0247 |
| M33 | 15.92 | [M-H]^-^ | 273.0085 | C_10_H_10_O_7_S | 274.01 | Ferulic acid+sulfation | 193.0511;178.0274;134.0369 |
| M34 | 16.47 | [M-H]^-^ | 369.0848 | C_16_H_18_O_10_ | 370.09 | Ferulic acid+glucuronidation | 193.0477;134.0342 |
| M35 | 16.49 | [M-H]^-^ | 387.0938 | C_16_H_20_O_11_ | 388.10 | Danshensu+Methylation+glucuronidation | 211.0645;167.0725 |
| M36 | 17.52 | [M-H]^-^ | 387.1311 | C_17_H_24_O_10_ | 388.14 | Loganin+deglycosylation+deoxidation+glucuronidation | 387.1298;327.1079;211.0980;165.0708;152.0486 |
| M37 | 17.59 | [M-H]^-^ | 369.0834 | C_16_H_18_O_10_ | 370.09 | Ferulic acid+glucuronidation | / |
| M38 | 18.70 | [M-H]^-^ | 387.1309 | C_17_H_24_O_10_ | 388.14 | Loganin+deglycosylation+deoxidation+glucuronidation | 387.1309;209.0832;152.0461 |
| M39 | 18.82 | [M-H]^-^ | 215.0017 | C_8_H_8_O_5_S | 216.01 | Caffeic acid-CO2+sulfation | 214.9982;135.0449 |
| M40 | 20.08 | [M-H]^-^ | 401.1096 | C_17_H_22_O_11_ | 402.12 | Morroniside+deglycosylation+dehydration+glucuronidation | 401.1087;225.0765;101.0244 |
| M41 | 20.47 | [M+H]^+^ | 401.1450 | C_18_H_24_O_10_ | 400.14 | Senkyunolide I+glucuronidation | 225.1118;210.0878;193.0856;165.0913;153.0539 |
| M42 | 21.62 | [M+H]^+^ | 401.1452 | C_18_H_24_O_10_ | 400.14 | Senkyunolide I+glucuronidation | 225.1124;210.0887;193.0854;165.0916 |
| M43 | 21.99 | [M-H]^-^ | 403.1268 | C_17_H_24_O_11_ | 404.13 | Morroniside+deglycosylation+dehydration+hydrogenation+glucuronidation | 403.1263;227.0906;127.0402 |
| M44 | 22.19 | [M+H]^+^ | 401.1465 | C_18_H_24_O_10_ | 400.14 | Senkyunolide H+glucuronidation | 225.1125;210.0886;193.0870;165.0912;150.0667 |
| M45 | 26.81 | [M-H]^-^ | 415.1265 | C_18_H_24_O_11_ | 416.13 | Morroniside+deglycosylation+dehydration+methylation+glucuronidation | 415.1284;224.0707;209.0438 |
| M46 | 28.58 | [M-H]^-^ | 381.1201 | C_18_H_22_O_9_ | 382.13 | Ligustilide+hydroxylation+glucuronidation | 381.1233;205.0863;187.0766 |
| M47 | 28.72 | [M+H]^+^ | 330.1387 | C_15_H_23_NO_5_S | 329.13 | SenkyunolideI+hydrogenation+cysteine conjugation | 330.1376;284.1326;191.1079;163.1114;153.0556 |
| M48 | 30.33 | [M+H]^+^ | 328.1231 | C_15_H_21_NO_5_S | 327.11 | Senkyunolide I+cysteine conjugation | / |
| M49 | 33.19 | [M-H]^-^ | 381.1215 | C_18_H_22_O_9_ | 382.13 | Ligustilide+hydroxylation+glucuronidation | 381.1194;205.0878 |
| M50 | 33.44 | [M+H]^+^ | 328.1227 | C_15_H_21_NO_5_S | 327.11 | Senkyunolide I+cysteine conjugation | 328.1233;282.1156;207.1018;189.0919 |
| M51 | 34.05 | [M+H]^+^ | 516.2037 | C_22_H_33_N_3_O_9_S | 515.19 | SenkyunolideI+hydrogenation+glutathione conjugation | 441.1729;387.1660;384.1247;162.0211 |
| M52 | 35.10 | [M+H]^+^ | 516.2023 | C_22_H_33_N_3_O_9_S | 515.19 | SenkyunolideH+hydrogenation+glutathione conjugation | 441.2128;387.1606;284.1348;162.0249 |
| M53 | 35.96 | [M-H]^-^ | 535.1106 | C_24_H_24_O_14_ | 536.12 | Rosmarinic acid+glucuronidation | 535.1074;359.0773;161.0244 |
| M54 | 37.31 | [M+H]^+^ | 514.1866 | C_22_H_31_N_3_O_9_S | 513.18 | Senkyunolide H+glutathione conjugation | 439.1583;385.1428;207.1050 |
| M55 | 37.79 | [M-H]^-^ | 381.1224 | C_18_H_22_O_9_ | 382.13 | Ligustilide+hydroxylation+glucuronidation | 381.1147;205.0868;161.0987 |
| M56 | 38.26 | [M+H]^+^ | 514.1905 | C_22_H_31_N_3_O_9_S | 513.18 | Senkyunolide I+glutathione conjugation | 514.1819;439.1523;385.1438;282.1167;207.1021 |
| M57 | 38.93 | [M-H]^-^ | 285.0431 | C_12_H_14_O_6_S | 286.05 | Ligustilide+hydroxylation+sulfation | 285.0423;205.0858;176.0475 |
| M58 | 41.72 | [M-H]^-^ | 549.1293 | C_25_H_26_O_14_ | 550.13 | Rosmarinic acid+methylation+glucuronidation | 549.2000;373.1754;175.0238 |
| M59 | 42.35 | [M-H]^-^ | 563.1451 | C_26_H_28_O_14_ | 564.15 | Rosmarinicacid+dimethylation+glucuronidation | 563.1412;387.1081;211.0612;175.0407 |
| M60 | 43.48 | [M-H]^-^ | 669.1491 | C_32_H_30_O_16_ | 670.15 | Salvianolic acid A+glucuronidation | / |
| M61 | 44.29 | [M-H]^-^ | 669.1493 | C_32_H_30_O_16_ | 670.15 | Salvianolic acid A+glucuronidation | 669.1476;493.1133;471.0917;295.0607;185.0235 |
| M62 | 44.71 | [M-H]^-^ | 563.1454 | C_26_H_28_O_14_ | 564.15 | Rosmarinicacid+dimethylation+glucuronidation | 563.1073;369.0827;193.0506;175.0243;149.0602 |
| M63 | 45.67 | [M-H]^-^ | 285.0450 | C_12_H_14_O_6_S | 286.05 | Ligustilide+hydroxylation+sulfation | 285.0422;205.0866;161.0970 |
| M64 | 47.14 | [M-H]^-^ | 467.0687 | C_20_H_20_O_11_S | 468.07 | Rosmarinic acid+dimethylation+sulfation | 467.0835;387.1048;211.0629;175.0427 |
| M65 | 48.50 | [M-H]^-^ | 551.1208 | C_28_H_24_O_12_ | 552.13 | Lithospermic acid+methylation | 551.1236;507.1321;327.0878;309.0775;294.0535;197.0456;179.0351 |
| M66 | 48.54 | [M-H]^-^ | 507.1313 | C_27_H_24_O_10_ | 508.14 | Salvianolic acid A+methylation | / |
| M67 | 48.76 | [M-H]^-^ | 683.1657 | C_33_H_32_O_16_ | 684.17 | SalvianolicacidA+methylation+glucuronidation | 683.1635;507.1364;471.0942;295.0603;185.0239 |
| M68 | 49.36 | [M-H]^-^ | 683.1674 | C_33_H_32_O_16_ | 684.17 | SalvianolicacidA+methylation+glucuronidation | 683.1647;507.1291;309.0772;294.0519;185.0204 |
| M69 | 49.48 | [M-H]^-^ | 565.1387 | C_29_H_26_O_12_ | 566.14 | Lithospermic acid+dimethylation | 565.2778;309.0789;211.0622 |
| M70 | 51.76 | [M-H]^-^ | 697.1831 | C_34_H_34_O_16_ | 698.18 | SalvianolicacidA+dimethylation+glucuronidation | 697.1804;521.1485;309.0785;211.0615 |
| M71 | 51.76 | [M-H]^-^ | 731.1639 | C_37_H_32_O_16_ | 732.17 | Salvianolic acid B+methylation | 731.1642;533.1141;353.0632;335.0602 |
| M72 | 53.59 | [M-H]^-^ | 697.1834 | C_34_H_34_O_16_ | 698.18 | SalvianolicacidA+dimethylation+glucuronidation | 697.1824;521.1514;309.0762;294.0523 |
| M73 | 54.30 | [M-H]^-^ | 697.1803 | C_34_H_34_O_16_ | 698.18 | SalvianolicacidA+dimethylation+glucuronidation | 697.1782;521.1517;309.0747;283.0972 |
| M74 | 54.59 | [M-H]^-^ | 565.1430 | C_29_H_26_O_12_ | 566.14 | Lithospermic acid+dimethylation | 565.1493;327.0913;309.0788;211.0624 |
| M75 | 55.26 | [M-H]^-^ | 565.1381 | C_29_H_26_O_12_ | 566.14 | Lithospermic acid+dimethylation | 309.0765;211.0613;193.0528 |
| M76 | 55.60 | [M+H]^+^ | 313.1459 | C_19_H_20_O_4_ | 312.14 | Neocryptotanshinone+hydrogenation | 313.1446;269.1567;253.0873;171.0823 |
| M77 | 56.20 | [M-H]^-^ | 697.1788 | C_34_H_34_O_16_ | 698.18 | SalvianolicacidA+dimethylation+glucuronidation | 697.1902;521.1459;309.0781;294.0542 |
| M78 | 61.63 | [M+H]^+^ | 293.0818 | C_18_H_12_O_4_ | 292.07 | Tanshinone I+hydroxylation | 283.0818;249.0897;193.1020;178.0785 |
