# Supplementary material for: Jiawei Kongsheng Zhenzhong Pill: marker compounds, absorption into the serum (rat), and Q-markers identified by UPLC-Q-TOF-MS/MS
Source: Front Pharmacol. 2024 Feb 5;15:1328632. doi: 10.3389/fphar.2024.1328632 (PMC10875140; doi:10.3389/fphar.2024.1328632)
Supplement: Supplementary file 2 [file Image1.pdf]

1.1 Supplementary Figures

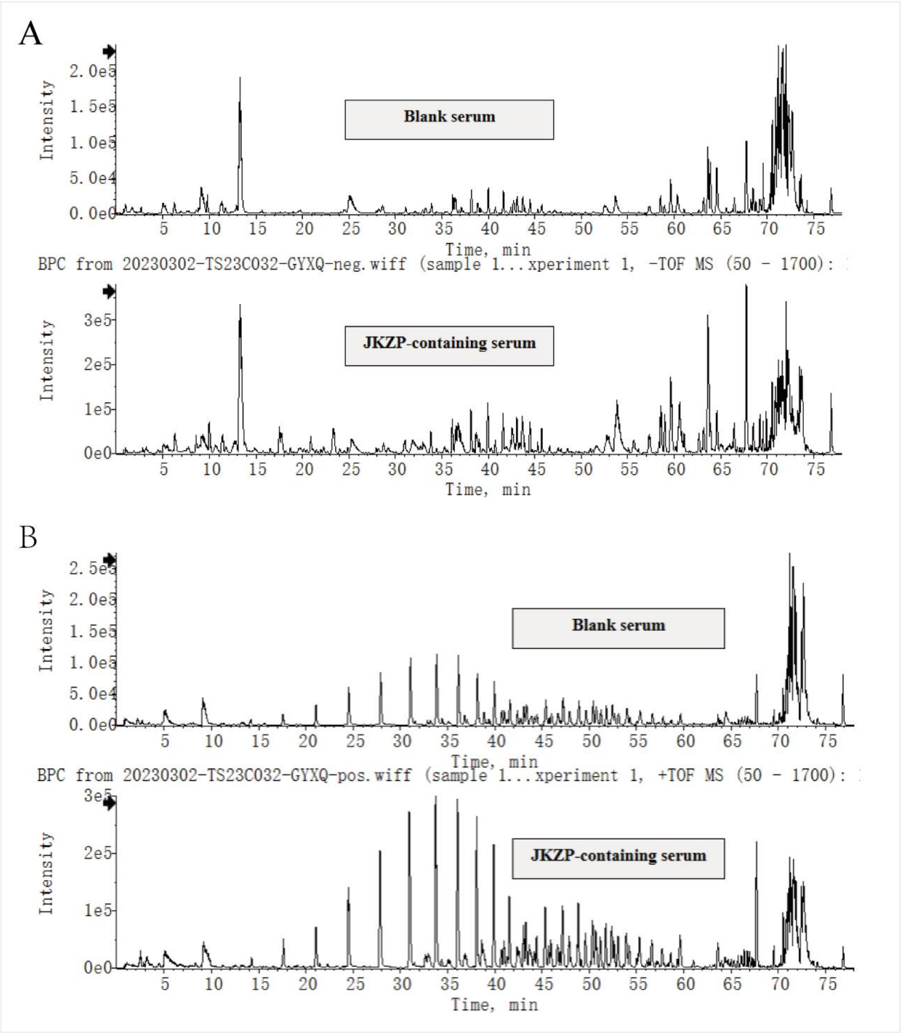

Supplementary Figure S1. Total ion flow diagram of JKZP-containing serum. (A) negative ion modes; (B) positive ion modes.

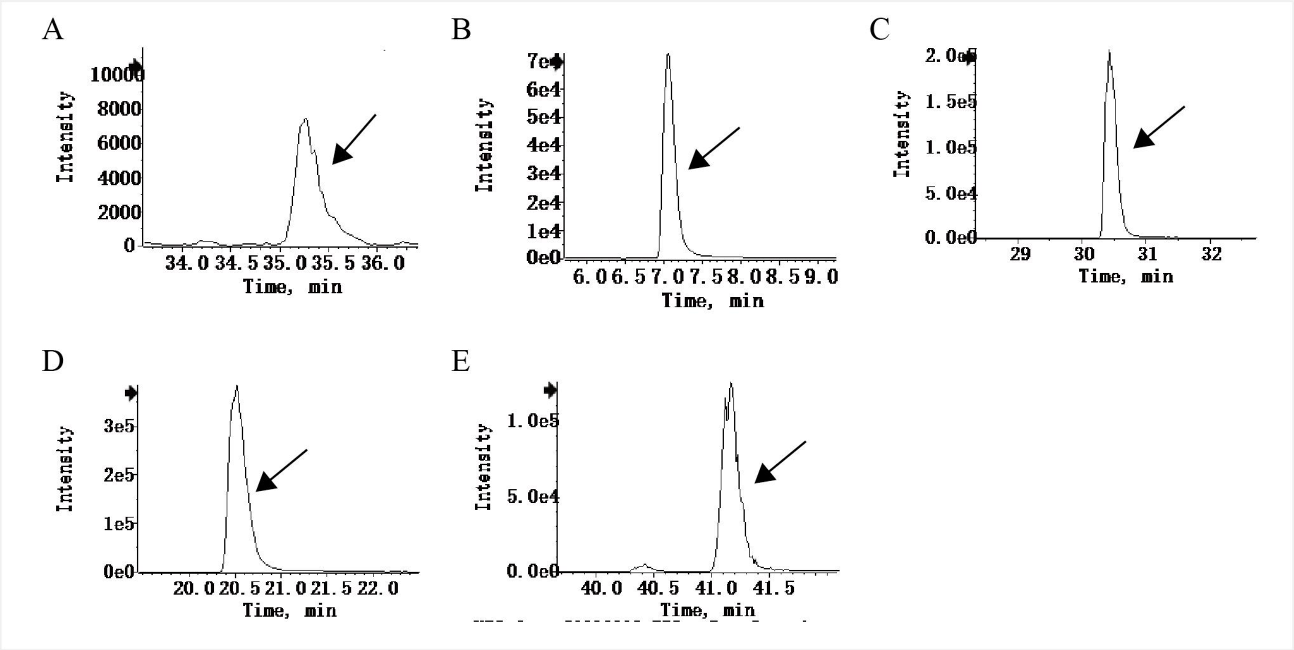

Supplementary Figure S2. (A-E) The maps of peaks in the JKZP aqueous extract of JKZP Q-marker. (A) Senkyunolide H; (B) Danshensu; (C) Echinacoside; (D) Loganic; (E) 3,6'-Disinapoyl sucrose.

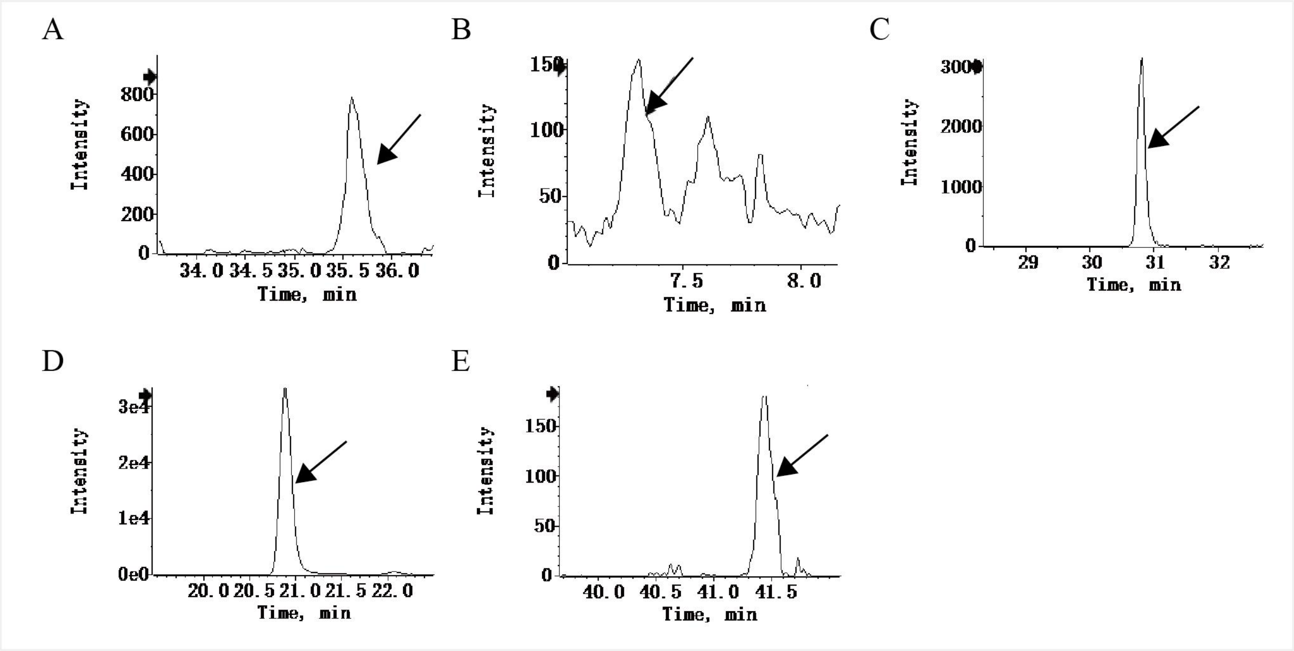

Supplementary Figure S3. (A-E) The maps of peaks in the JKZP-containing serum of JKZP Q-marker. (A) Senkyunolide H; (B) Danshensu; (C) Echinacoside; (D) Loganic; (E) 3,6’-Disinapoyl sucrose.
